# Prognostic perspectives of PD-L1 combined with tumor-infiltrating lymphocytes, Epstein-Barr virus, and microsatellite instability in gastric carcinomas

**DOI:** 10.1186/s13000-020-00979-z

**Published:** 2020-06-04

**Authors:** Euno Choi, Mee Soo Chang, Sun-ju Byeon, Heejin Jin, Kyeong Cheon Jung, Haeryoung Kim, Kook Lae Lee, Won Kim, Jin Hyun Park, Ki Hwan Kim, Jin-Soo Kim, In Sil Choi, Dong-Seok Han, Hye Seong Ahn, Seung Chul Heo

**Affiliations:** 1 Department of Pathology, Seoul National University Boramae Hospital, Seoul National University College of Medicine, 20 Boramae-ro 5-gil, Dongjak-gu, Seoul, 07061 Republic of Korea; 2 Medical Research Collaborating Center, Department of Biostatistics, Seoul National University Boramae Hospital, Seoul, Republic of Korea; 3 Department of Pathology, Seoul National University Hospital, Seoul National University College of Medicine, Seoul, Republic of Korea; 4 Department of Internal Medicine, Seoul National University Boramae Hospital, Seoul National University College of Medicine, Seoul, Republic of Korea; 5 Department of Surgery, Seoul National University Boramae Hospital, Seoul National University College of Medicine, Seoul, Republic of Korea

**Keywords:** Stomach cancer, PD-L1, Tumor-infiltrating lymphocytes, Epstein-Barr virus, Microsatellite instability, Prognosis

## Abstract

**Background:**

The prognostic potential of PD-L1 is currently unclear in gastric carcinomas, although the immune checkpoint PD-1/PD-L1 inhibitors have produced promising results in clinical trials.

**Methods:**

We explored the prognostic implications of programmed death ligand 1 (PD-L1) in 514 consecutive surgically-resected gastric carcinomas. Overall survival and recurrence-free survival were evaluated. Immunohistochemistry for PD-L1, CD8, FOXP3, and PD-1, and molecular grouping by in situ hybridization for Epstein-Barr virus (EBV)-encoded small RNAs and multiplex PCR for microsatellite instability (MSI) markers were performed. Additionally, to explore the function inherent to PD-L1, PD-L1-specific siRNA transfection, cell proliferation, invasion, migration and apoptosis assays were conducted in five gastric carcinoma cell lines.

**Results:**

PD-L1(+) tumor and immune cells were observed in 101 (20%) and 244 patients (47%), respectively. “Tumoral PD-L1(+)/immune cell PD-L1(-)/CD8^+/low^ tumor-infiltrating lymphocytes (TILs),” and more advanced-stage tumors were associated with unfavorable clinical outcomes in the entire cohort through multivariate analysis. Furthermore, tumoral PD-L1(+)/FOXP3^+/low^ TILs were associated with worse clinical outcomes in EBV-positive and MSI-high carcinomas. Tumoral PD-L1(+) alone was an adverse prognostic factor in EBV-positive carcinomas, but not in MSI-high carcinomas, whereas PD-L1(+) immune cells or FOXP3^+/high^ TILs alone were correlated with a favorable prognosis. PD-L1 knockdown in gastric carcinoma cells suppressed cell proliferation, invasion and migration, and increased apoptosis, which were all statistically significant in two EBV(+) cell lines, but not all in three EBV(−) cell lines.

**Conclusions:**

The prognostic impact of PD-L1 may depend on the tumor microenvironment, and statuses of EBV and MSI, although PD-L1 innately promotes cancer cell survival in cell-based assays. The combination of “tumoral PD-L1/immune cell PD-L1/CD8^+^ TILs” may serve as an independent prognostic factor. Tumoral PD-L1(+)/immune cell PD-L1(−)/CD8^+/low^ TILs showing a worse prognosis may be beneficial for combinatorial therapies of anti-PD-L1/PD-1 and anti-cytotoxic T-lymphocyte associated antigen 4 (CTLA4) that would promote effector T cells, thus attack the tumor.

## Background

Gastric carcinoma is the third most common cancer, and the third or fourth most common cause of cancer-related deaths worldwide [1]. Recently, targeting programmed cell death 1 (PD-1)/programmed cell death ligand 1 (PD-L1) immune checkpoint inhibitors have led to major progress in cancer immunotherapy; resulting in positive outcomes in clinical trials across various solid malignancies, including gastric carcinomas [2, 3]. Tumor cells aberrantly express PD-L1, and exploit PD-1/PD-L1 molecular brakes to evade immune surveillance [4, 5]. Binding of the transmembrane PD-1 protein to its ligand, PD-L1, results in PD-1/T cell reoceptor inhibitory micro-clusters, which suppress the activation of T cells that could otherwise attack tumor cells [4].

The tumor microenvironment contains tumor cells that evade immunity by reprogramming immune cells, resulting in a dynamic immune environment [6]. Tumor-infiltrating lymphocytes (TILs), particularly, CD8^+^ cytotoxic T cells, support tumor cell killing functions [7, 8]. However, their prolonged exposure to cancer cells may lead to the loss of their effector function [8]. Furthermore, PD-L1 promotes the initiation, maintenance, and expansion of forkhead box P3 (FOXP3)^+^ regulatory T cells (Treg), which inhibit antitumor CD8^+^ cytotoxic T cell functions [9]. Thus, immunomodulatory TILs may play an important role in the action of immune checkpoint blockades [10].

Many studies have investigated the prognostic potential of PD-L1 expression, but their data are controversial [11]; poor [12–15], good [16], and neutral [17] prognostic outcomes have all been reported. Given the convoluted immune interactions that occur in the tumor microenvironment, a combinatorial analysis of PD-L1, TILs, Epstein-Barr virus (EBV)-infection, and microsatellite instability (MSI) status is required. However, very few studies have employed such an integrative analysis.

In this study, we investigated PD-L1 expression in tumor and stromal immune cells, TILs (CD8^+^, FOXP3^+^, and PD-1^+^ cells), and their concomitant prognostic value in a large cohort of gastric carcinomas and in molecular groups stratified by EBV-infection and MSI status. We aimed to define the prognostic implications of PD-L1 and immunomodulatory TILs, and further, provide guidance regarding the selection of patients for whom PD-1/PD-L1 blockade immunotherapy could be advantageous.

## Methods

### Patients

We collected 514 surgically resected gastric carcinomas at the Seoul National University Boramae Hospital (Seoul, Korea) between 2006 and 2011. After surgical resection, patients in tumor stage II received adjuvant chemotherapy with 5-fluorouracil (5-FU) or 5-FU/mitomycin-C, and patients in stage III or IVA, with 5-FU/cisplatin. None of patients were treated with neoadjuvant chemotherapy or immunotherapy. We reviewed medical records, patient outcomes, and histopathological findings, such as the World Health Organization (WHO) histologic classification, Lauren histologic type [18], and the tumor stage (pathological tumor-node-metastasis (pTNM)) based on the 7th American Joint Committee on Cancer [19]. Overall survival or recurrence-free survival was estimated from the date of surgery to death, tumor recurrence, or the last follow-up visit. The median follow-up period for overall survival was 77 months (mean: 63.8, range: ~ 1–128).

### Generating tissue microarray blocks

After a histological review of all tumor sections, the portion of deepest tumor invasion was chosen from each donor block, and two tissue cores (diameter: 2 mm/core) per tumor were punched out using a trephine. The tissue cores were then inserted in a new recipient block containing fifty-nine tissue cores and one ink core as a direction marker. A total of eighteen tissue microarray blocks were thus prepared for immunohistochemistry.

### Immunohistochemistry for PD-L1, CD8, FOXP3, and PD-1

Immunohistochemistry was performed using an automated immunostainer, the BenchMark Ultra IHC/ISH system (Ventana Medical Systems, Tucson, AZ, USA), according to the manufacturer’s protocol. For PD-L1 immunohistochemistry, two different antibodies were utilized: clone E1L3N® (1:30, Cell Signaling Technology, Danvers, MA, USA) was used for tumoral PD-L1 evaluation, and SP263 (Ready to use, Ventana Medical Systems) was used for stromal immune cell PD-L1 detection; two different PD-L1 antibodies were used because they have been shown to have different efficiencies for the detection of tumoral PD-L1 and stromal immune cell PD-Ll [20]. We additionally conducted immunohistochemistry for CD8 (Ready to use; Novocastra, Leica Microsystems, Wetzlar, Germany), FOXP3 (236A/E7, 1:30; Abcam, Cambridge, MA, USA), and PD-1 (NAT105, 1:30; Cell Marque, Rocklin, CA, USA).

PD-L1 expression in tumor cells was primarily scored based on staining intensity and percentage of stained tumor cells; any membranous staining was regarded as “positive expression” [21]. Stromal immune cell PD-L1 was categorized as positively expressed when membranous staining was present in ≥5% of the stromal immune cells at any staining intensity [22]. The number of CD8-, FOXP3-, or PD-1-positive cells were counted in ten contiguous high-power fields in heavily infiltrated areas; the absolute number of immunostained cells in each group was determined as an average per high-power field (400× magnification, 0.24 mm^2^) (Olympus BX51 microscope; Olympus, Tokyo, Japan). Finally, groups were classified into “low” and “high” populations based on the median number of CD8^+^, FOXP3^+^, and PD-1^+^ TILs cells, and then denoted as ^+/low^ or ^+/high^ [23].

### In situ hybridization for EBV-encoded small RNAs

In situ hybridization for EBV-encoded small RNAs (EBER) was conducted using the BenchMark Ultra IHC/ISH system and the INFORM EBER probe (Ventana Medical Systems) according to the manufacturer’s instructions. EBV-infected cells were observed as black-colored signals at the hybridization site using light microscopy. Only signals within tumor cell nuclei were considered EBV-positive carcinomas; black signals were seen in almost all cancer cell nuclei in EBV-positive cases.

### Microsatellite instability analysis

Immunohistochemical staining for human mutL homolog 1 (hMLH1; Ready to use; Ventana Medical Systems) and human mutS homolog 2 (hMSH2; Ready to use; Cell Marque) was initially performed on full-section paraffin blocks to screen for MSI-high cases, as reported previously [24]. Next, in cases showing loss patterns for either hMLH1 or hMSH2 nuclear expression, we extracted the DNA from paired normal and tumor tissues, and subsequently carried out MSI analysis using fluorescent multiplex PCR with five markers (BAT-25, BAT-26, D5S346, D17S250, and D2S123), as recommended by the National Cancer Institute (NCI) [25]. The PCR products were analyzed with a DNA autosequencer (ABI 3731 Genetic Analyzer; Applied Biosystems, Foster City, CA, USA). When a case was positive for two or more microsatellite markers, it was defined as MSI-high, in accordance with NCI criteria [25].

### PD-L1 innate function study in gastric carcinoma cell lines

We purchased SNU601, SNU216, and SNU719 from the Korean Cell Line Bank (Seoul, Korea), and AGS, from the American Type Culture Collection (Manassas, VA, USA). YCCEL1 was supplied by Dr. SY Rha [26, 27]. Cells were maintained at 37 °C in RPMI 1640 (Gibco BRL, Rockville, MD, USA) supplemented with 10% fetal bovine serum, 2 mmol/L L-glutamine, and antibiotics (100 units/mL penicillin and streptomycin) in a humidified 5% CO_2_/95% air atmosphere.

PD-L1-specific siRNA (s26547; 5′-GGCAUUUGCUGAACGCAUU-3′) was acquired from Ambion Applied Biosystems (Austin, TX, USA), and a scrambled siRNA (sc-37,007) as the negative control, from Santa Cruz Biotechnology (Santa Cruz, CA, USA). We transfected cells (25 × 10^4^ in a 60 mm plate) with 200 pmol siRNA using Lipofectamine 2000 (Invitrogen) per manufacturer’s instructions. After 24 h, western blot was performed; protein separated by SDS-PAGE gel was transferred onto PVDF membranes (Millipore, Bedford, MA, USA). Each membrane was treated with the primary antibody, PD-L1 (17952–1-AP, 1:1000, Proteintech Fisher Scientific: Hampton, NH, USA), and then secondary antibodies (Cell Signaling). As a loading and transfer control, we used an antibody against β-actin (AC-15, 1:10000, Abcam).

To examine cellular proliferation, cells were seeded into 96-well plates (10^3^ cells/well) overnight, and incubated with 100 μL/well of Cell Counting Kit-8 (CCK-8; Dojindo Laboratories, Kumamoto, Japan) for 2 h in the dark. Absorbance at 450 nm was measured using a spectrophotometer (Spectramax 190; Molecular Devices, Sunnyvale, CA, USA).

As for cell invasion, 24-well BioCoat Matrigel invasion chambers (BD Biosciences, San Diego, CA, USA) was used. Cells (5 × 10^4^ /well in a 24-well plate) were placed in the upper chamber filled with 500 μL serum-free media, and the lower chamber was filled with 700 μL media supplemented with 10% fetal bovine serum; the chambers were then incubated for 18 h at 37 °C. Then, cells that invaded the lower chamber were stained with 4 μg/mL Calcein AM (BD Biosciences) in Hank’s buffered saline at 37 °C for 1 h, and counted on a fluorescence microscope (Olympus IX71, Tokyo, Japan).

Apoptosis was calibrated with the Annexin V-FITC apoptosis kit (BD Biosciences). Cells (10 × 10^4^/well in a 6-well plate) were cultured in serum-free media, trypsinized (Invitrogen, Carlsbad, CA, USA), centrifuged, and re-suspended in Annexin V–binding buffer (150 mmol/L NaCl, 18 mmol/L CaCl_2_, 10 nmol/L HEPES, 5 mmol/L KCl, and 1 mmol/L MgCl_2_). Cells were incubated with FITC-conjugated Annexin V (1 μg/mL) and propidium iodide (50 μg/mL) for 30 min at room temperature in the dark, and analyzed with FACScan flow cytometer (Becton Dickinson, Mountain View, CA, USA) and CellQuest software (Becton Dickinson).

Wound healing assay was performed to assess cell migration. A scratch was made in a 6-well plate of confluent cells (25 × 10^4^/well) with the tip of a micropipette. Images were taken 24 h later on an inverted photomicroscope (Olympus IX71). Movements of individual cells were measured with NIH ImageJ software (http://rsb.info.nih.gov/ij/index.html).

### Statistical analysis

Pearson’s chi-squared test, two-tailed Student’s t-test, Spearman’s rank correlation coefficient, and Mann-Whitney U tests were used. Patient survival rates were calculated using the Kaplan-Meier method and log-rank test. We inputted parameters for which the *P* value < 0.05 in univariate analysis into a Cox proportional hazard model (multivariate analysis) to calculate a hazard ratio (HR) and 95% confidence interval (CI). A value of *P* <  0.05 was considered statistically significant. All statistical analyses were conducted in SPSS Statistics version 21.0 for Windows (IBM SPSS Inc., Armonk, NY, USA) or the R Project for Statistical Computing 3.6.3 (https://cran.r-project.org/bin/windows/base/).

## Results

### Clinicopathological features, PD-L1 expression, and TILs in the entire cohort

**Fig. 1:**
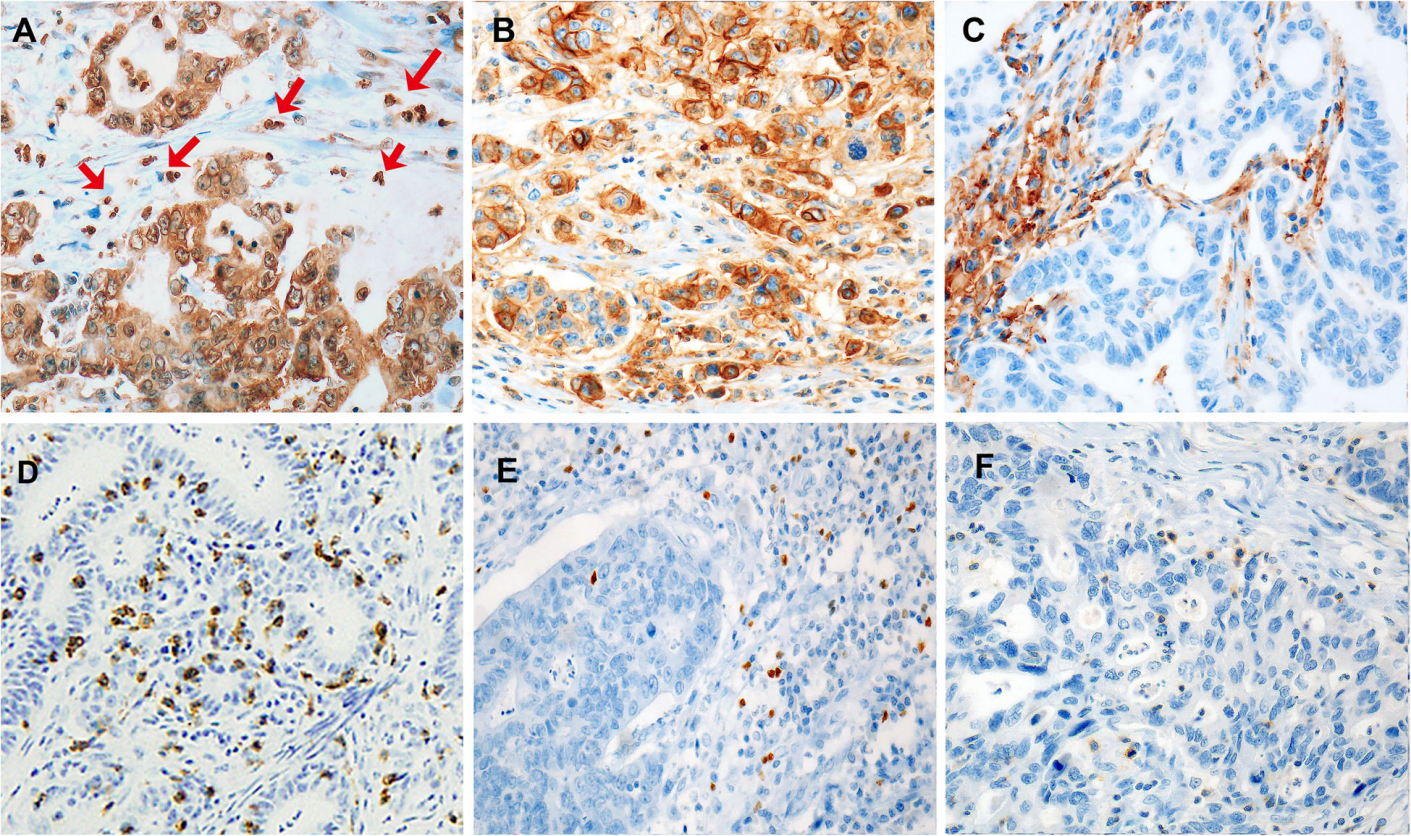
Immunohistochemical features of PD-L1, CD8^+^, FOXP3^+^, and PD-1^+^. (A-C) PD-L1 is expressed in three major patterns: **a** “Focal” PD-L1 expression in tumor cells and immune cells (red arrows). **b** “Diffuse (positive ≥10% of tumor cells)” PD-L1 expression in tumor cells and no staining in immune cells. **c** PD-L1 expression in immune cells only. (D-F) Note the high population in each line of tumor-infiltrating lymphocytes (TILs); **d** CD8^+/high^ TILs, **e** FOXP3^+/high^ TILs, and **f** PD-1^+/high^ TILs

PD-L1(+) tumor cells were observed in 101 (20%) out of 514 cases, and PD-L1(+) immune cells were observed in 244 cases (47%) (Table 1 and Fig. 1). The dual expression of PD-L1 in both tumor and immune stromal cells was observed in 65 cases (13%). The tumoral or immune cell PD-L1(+) subgroup manifested many more CD8^+^, FOXP3^+^, and PD-1^+^ TILs than the tumoral PD-L1(−) or immune cell PD-L1(−) subgroup (*P* <  0.05, respectively) (Supplemental Table 1 and Supplemental Fig. 1).

**Table 1:** Relationship Between Clinicopathological Features and PD-L1 Expression and Tumor-Infiltrating Lymphocytes in the Entire Cohort

	Total (*N* = 514)	PD-L1 in tumor cells			PD-L1 in immune cells		
		positive		negative	positive	negative	
Sex							
Male	347 (68%)	65 (19%)		282 (81%)	170 (49%)	177 (51%)	
Female	167 (32%)	36 (22%)		131 (78%)	74 (44%)	93 (56%)	
Age, median (years, range)	65 (27–88)	66 (27–88)		64 (29–88)	66 (27–88)	64 (30–86)	
Tumor site							
Lower 1/3	332 (65%)	63 (19%)		269 (81%)	156 (47%)	176 (53%)	
Middle 1/3	103 (20%)	17 (17%)		86 (83%)	50 (49%)	53 (51%)	
Upper 1/3	79 (15%)	21 (27%)		58 (73%)	38 (48%)	41 (52%)	
Histologic type		* *P* = 0.008			* *P* < 0.001		
Tubular adenocarcinoma	415 (81%)	87 (21%)		328 (79%)	214 (52%)	201 (48%)	
Poorly cohesive carcinoma	83 (16%)	10 (12%)		73 (88%)	22 (27%)	61 (73%)	
Mucinous carcinoma	9 (2%)	0		9 (100%)	2 (22%)	7 (78%)	
Undifferentiated carcinoma	7 (1%)	4 (57%)		3 (43%)	6 (86%)	1 (14%)	
Lauren classification		* *P* = 0.023			* *P* = 0.019		
Intestinal	286 (56%)	46 (16%)		240 (84%)	149 (52%)	137 (48%)	
Diffuse	228 (44%)	55 (24%)		173 (76%)	95 (42%)	133 (58%)	
Lymphatic invasion		* *P* < 0.001					
Present	230 (45%)	67 (29%)		163 (71%)	100 (43%)	130 (57%)	
Depth of invasion (pT)		* *P* < 0.001			* *P* = 0.006		
pT1 (mucosa, submucosa)	262 (51%)	29 (11%)		233 (89%)	137 (52%)	125 (48%)	
pT2 (muscularis proper)	46 (9%)	10 (22%)		36 (78%)	27 (59%)	19 (41%)	
pT3 (subserosa)	97 (19%)	25 (26%)		72 (74%)	42 (43%)	55 (57%)	
pT4 (serosa or beyond)	109 (21%)	37 (34%)		72 (66%)	38 (35%)	71 (65%)	
Lymph node metastasis		* *P* = 0.008			* *P* = 0.004		
Present	215 (42%)	54 (25%)		161 (75%)	86 (40%)	129 (60%)	
Tumor stage (pTNM)		* *P* < 0.001			* *P* = 0.008		
Stage I	277 (54%)	36 (13%)		241 (87%)	148 (53%)	129 (47%)	
Stage II	81 (16%)	23 (28%)		58 (72%)	39 (48%)	42 (52%)	
Stage III	130 (25%)	33 (25%)		97 (75%)	49 (38%)	81 (62%)	
Stage IV	26 (5%)	9 (35%)		17 (65%)	8 (31%)	18 (69%)	
	Total (*N* = 514)	CD8^+^ population		FOXP3^+^ population		PD-1^+^ population	
		high	low	high	low	high	low
Sex							
Male	347 (68%)	177 (51%)	170 (49%)	161 (46%)	186 (54%)	126 (36%)	221 (64%)
Female	167 (32%)	80 (48%)	87 (52%)	79 (47%)	88 (53%)	55 (33%)	112 (67%)
Age, median (years, range)	65 (27–88)	65 (27–88)	65 (30–86)	66 (27–84)	63 (29–88)	67 (40–88)	63 (27–88)
Tumor site						* P = 0.049	
Lower 1/3	332 (65%)	162 (49%)	170 (51%)	162 (49%)	170 (51%)	108 (33%)	224 (67%)
Middle 1/3	103 (20%)	46 (45%)	57 (55%)	46 (45%)	57 (55%)	37 (36%)	66 (64%)
Upper 1/3	79 (15%)	49 (62%)	30 (38%)	32 (41%)	47 (59%)	36 (46%)	43 (54%)
Histologic type				* P = 0.001			
Tubular adenocarcinoma	415 (81%)	209 (50%)	206 (50%)	209 (50%)	206 (50%)	152 (37%)	263 (63%)
Poorly cohesive carcinoma	83 (16%)	37 (45%)	46 (55%)	25 (30%)	58 (70%)	22 (27%)	61 (73%)
Mucinous carcinoma	9 (2%)	4 (44%)	5 (56%)	3 (33%)	6 (67%)	1 (11%)	8 (89%)
Undifferentiated carcinoma	7 (1%)	7 (100%)	0	3 (43%)	4 (57%)	6 (86%)	1 (14%)
Lauren classification		* P = 0.004		* P = 0.002			
Intestinal	286 (56%)	127 (44%)	159 (56%)	151 (53%)	135 (47%)	97 (34%)	189 (66%)
Diffuse	228 (44%)	130 (57%)	98 (43%)	89 (39%)	139 (61%)	84 (37%)	144 (63%)
Lymphatic invasion		* P = 0.033		* P = 0.017		* P = 0.041	
Present	230 (45%)	127 (55%)	103 (45%)	94 (41%)	136 (59%)	92 (40%)	138 (60%)
Depth of invasion (pT)		* P = 0.006		* P < 0.001			
pT1 (mucosa, submucosa)	262 (51%)	112 (43%)	150 (57%)	152 (58%)	110 (42%)	85 (32%)	177 (68%)
pT2 (muscularis proper)	46 (9%)	31 (67%)	15 (33%)	22 (48%)	24 (52%)	20 (43%)	26 (57%)
pT3 (subserosa)	97 (19%)	54 (56%)	43 (44%)	34 (35%)	63 (65%)	34 (35%)	63 (65%)
pT4 (serosa or beyond)	109 (21%)	60 (55%)	49 (45%)	32 (29%)	77 (71%)	42 (39%)	67 (61%)
Lymph node metastasis				* *P* < 0.001			
Present	215 (42%)	112 (52%)	103 (48%)	71 (33%)	144 (67%)	77 (36%)	138 (64%)
Tumor stage (pTNM)				* P < 0.001			
Stage I	277 (54%)	126 (45%)	151 (55%)	163 (59%)	114 (41%)	95 (34%)	182 (66%)
Stage II	81 (16%)	50 (62%)	31 (38%)	29 (36%)	52 (64%)	30 (37%)	51 (63%)
Stage III	130 (25%)	66 (51%)	64 (49%)	39 (30%)	91 (70%)	44 (34%)	86 (66%)
Stage IV	26 (5%)	15 (58%)	11 (42%)	9 (35%)	17 (65%)	12 (46%)	14 (54%)

PD-L1 positive in tumor cells (*n* = 101); PD-L1 negative in tumor cells (*n* = 413); PD-L1 positive in immune cells (*n* = 244); PD-L1 negative in immune cells (*n* = 270)

CD8^+^ high (*n* = 257), CD8^+^ low (*n* = 257); FOXP3^+^ high (*n* = 240); FOXP3^+^ low (*n* = 274), PD-1^+^ high (*n* = 181); PD-1^+^ low (*n* = 333)

*P* values with statistically significant differences (< 0.05) are marked with an asterisk (*)

### Prognostic value of PD-L1 expression, TILs, EBV-infection, and MSI status in the entire cohort

**Fig. 2:**
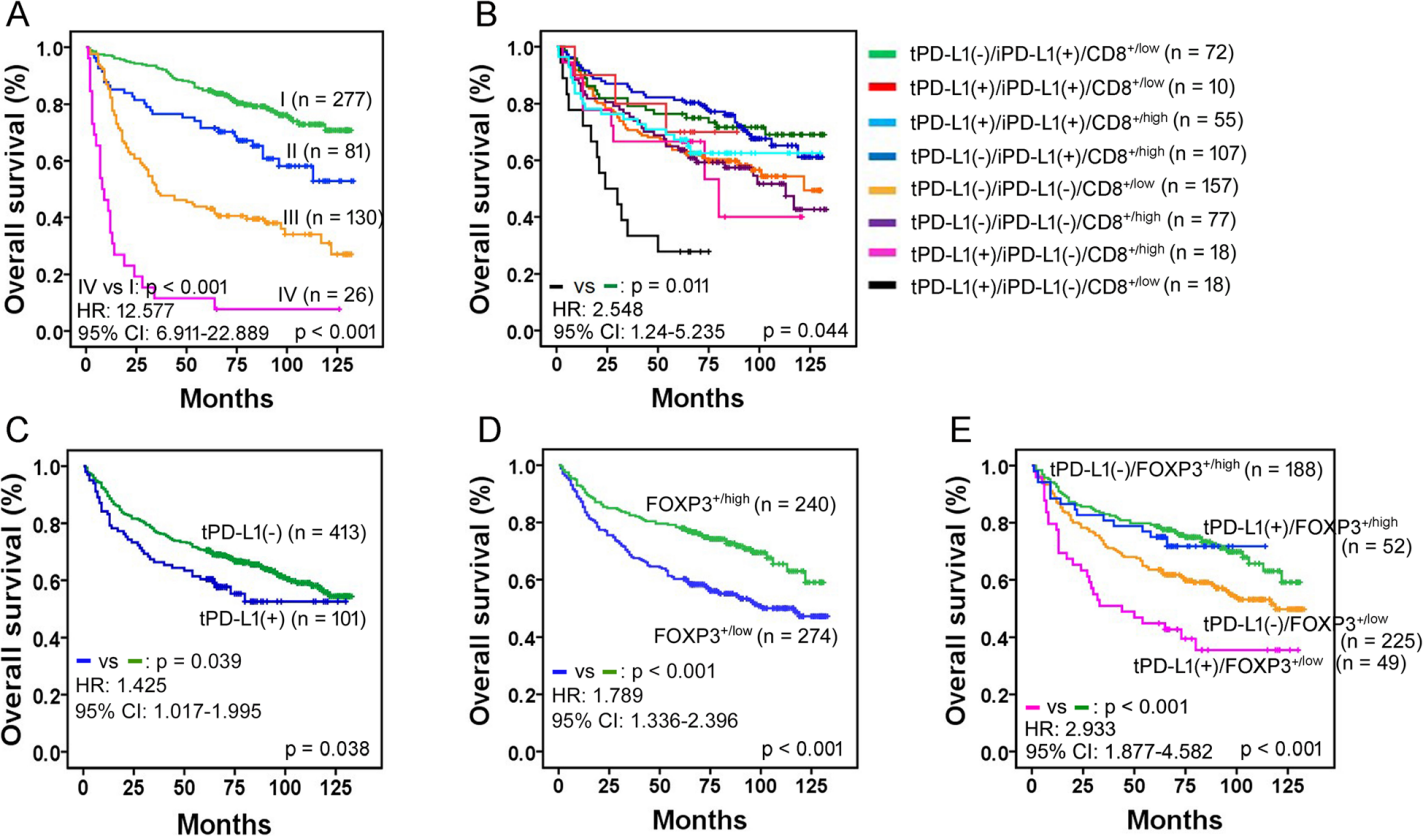
Kaplan-Meier survival plots of overall survival. In the entire cohort (*N* = 514), the subgroups with more advanced-stage tumors (**a**), the combined subset of tumoral PD-L1(+)/immune cell PD-L1(−)/CD8^+low^ tumors (**b**) exhibit the lowest rates of patient survival within each corresponding analysis based on the Cox proportional hazards model. I, II, III and IV in (A) indicate TNM tumor stage. Tumoral PD-L1(+) (**c**), FOXP3^+/low^ (**d**), and the combined subset of tumoral PD-L1(+)/FOXP3^+/low^ tumors (**e**) reveal the lowest rates of patient survival through each corresponding univariate analysis. Corresponding *P* value, hazard ratio (HR) and 95% confidence interval (CI) in the worst prognostic subset are shown in the bottom left corner of each plot, and *P* values throughout all subsets, in the bottom right corner of each plot

The subgroup of more advanced-stage tumors (pTNM), Lauren diffuse type, presence of lymphatic invasion, tumoral PD-L1(+), immune cell PD-L1(−), or FOXP3^+/low^ TILs was associated with lower rates of overall survival via univariate analysis of the cohort (*N* = 514) (Fig. 2 and Supplemental Table 2). Since most patients with tumor recurrence did not survive, the above mentioned factors were correlated with lower rates of recurrence-free survival (data not shown).

In our combined analysis of tumoral PD-L1 and each subtype of TILs, the combined subsets of tumoral PD-L1(+)/CD8^+/low^ TILs, tumoral PD-L1(+)/immune cell PD-L1(−), or tumoral PD-L1(+)/FOXP3^+/low^ TILs showed a worse clinical outcome based on univariate analysis (*P* <  0.05, respectively). In combined analysis of three components from tumoral PD-L1, immune cell PD-L1, and each subtype of TILs, the combined subset of tumoral PD-L1(+)/immune cell PD-L1(−)/CD8^+/low^ TILs was related with an adverse clinical outcome (*P* <  0.05).

Multivariate analysis showed that an unfavorable prognosis was maintained in more advanced-stage tumors, the subset of tumoral PD-L1(+)/CD8^+/low^ TILs, or the subset of tumoral PD-L1(+)/immune cell PD-L1(−)/CD8^+/low^ TILs, and all were independent prognostic factors (*P* < 0.05, each) (Fig. 2 and Supplemental Table 2). EBV or MSI status alone were not a prognostic factor in the cohort.

Our analysis of only patients with “advanced gastric carcinoma” (AGC; cases with tumor invasion into the proper muscle or deeper; *N* = 253) revealed that the prognostic factors for AGC were the same as those for the entire cohort, with two exceptions: AGC patients with a low population of CD8^+^ TILs showed lower rates of overall survival and recurrence-free survival through univariate analysis, and the MSI-high AGC group showed a more favorable clinical outcome (*P* < 0.05) (Supplemental Fig. 2).

### Clinicopathological features, PD-L1, and TILs in EBV-positive and MSI-high gastric carcinomas

**Fig. 3:**
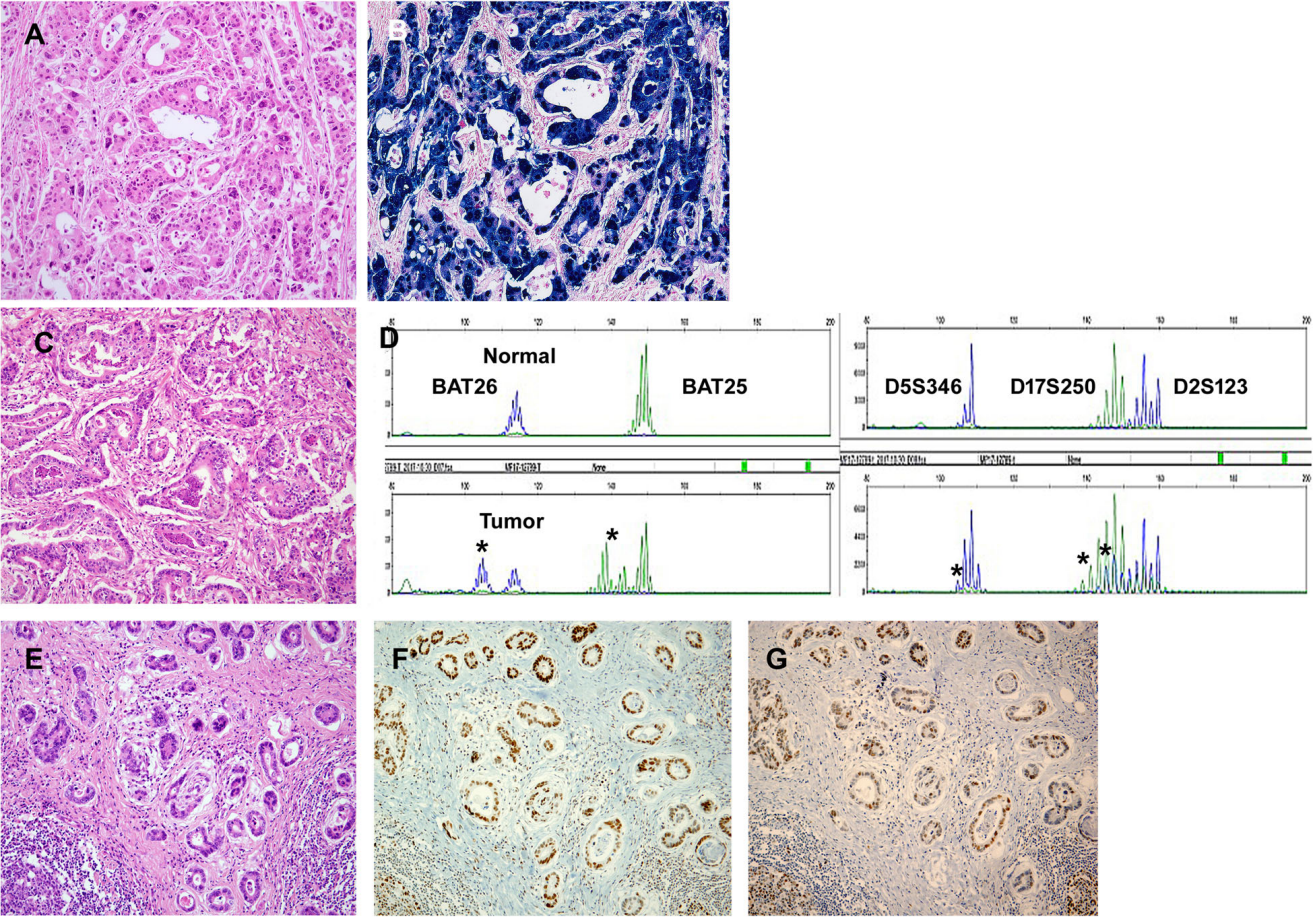
Representative features of each molecular group. **a**, **c**, **e** Hematoxylin and eosin-stained images of each group. **a**-**b** Epstein-Barr virus (EBV)-positive gastric carcinoma; **b** Black signals are detected in most tumor cell nuclei by in situ hybridization for EBV-encoded small RNAs. **c**-**d** Microsatellite instability (MSI)-high gastric carcinoma; **d** Microsatellites are recognized for five markers (BAT26, BAT25, D5S346, D17S250, and D2S123) by multiplex PCR, and are indicated with asterisks (*). **e**-**g** Conventional carcinoma (EBV-negative and non-MSI-high); hMLH1 **f** and hMSH2 **g** were retained in tumor cell nuclei

Of the 514 gastric carcinoma patients, there were 32 (6%) and 53 (10%) cases of EBV-positive and MSI-high gastric carcinomas, respectively; No patients were both EBV-positive and MSI-high. The remaining 429 cases of EBV-negative/non-MSI-high cases were classified as conventional gastric carcinomas (Fig. 3). Unlike the conventional group, EBV-positive carcinomas were located predominantly in the upper 1/3 of the stomach and were more commonly Lauren diffuse type (*P* < 0.05 for each). Additionally, MSI-high carcinomas showed more frequent lymphatic invasion, deeper invasion depth, and more advanced-stage tumors (*P* < 0.05) (Table 2).

**Table 2:** Clinicopathological Features in Each Molecular Group

	Conventional	EBV-positive	MSI-high
	(*n* = 429)	(*n* = 32)	(*n* = 53)
Sex			
Male	290 (68%)	24 (75%)	33 (62%)
Female	139 (32%)	8 (25%)	20 (38%)
Age, median (years, range)	65 (27–88)	63 (45–88)	64 (34–82)
Tumor site		*^a^*P* < 0.001	
Lower 1/3	287 (67%)	4 (13%)	41 (77%)
Middle 1/3	85 (20%)	11 (34%)	7 (13%)
Upper 1/3	57 (13%)	17 (53%)	5 (9%)
Histologic type			*^b^*P* = 0.006
Tubular adenocarcinoma	343 (80%)	29 (91%)	43 (81%)
Poorly cohesive	76 (18%)	2 (6%)	5 (9%)
Mucinous carcinoma	5 (1%)	0	4 (8%)
Undifferentiated	5 (1%)	1 (3%)	1 (2%)
Lauren		*^a^*P* = 0.029	
Intestinal	246 (57%)	12 (38%)	28 (53%)
Diffuse	183 (43%)	20 (63%)	25 (47%)
Lymphatic invasion			*^b^*P* < 0.001
Present	182 (42%)	11 (34%)	37 (70%)
Depth of invasion (pT)			*^b^*P* = 0.031
pT1 (mucosa, submucosa)	229 (53%)	17 (53%)	16 (30%)
pT2 (muscularis proper)	37 (9%)	3 (9%)	6 (11%)
pT3 (subserosa)	79 (18%)	2 (6%)	16 (30%)
pT4 (serosa or beyond)	84 (20%)	10 (31%)	15 (28%)
Lymph node metastasis			
Present	177 (41%)	10 (31%)	28 (53%)
Tumor stage (pTNM)			*^b^*P* = 0.028
Stage I	238 (55%)	20 (63%)	19 (36%)
Stage II	64 (15%)	3 (9%)	14 (26%)
Stage III	105 (24%)	7 (22%)	18 (34%)
Stage IV	22 (5%)	2 (6%)	2 (4%)

*EBV* Epstein-Barr virus, *MSI* Microsatellite instability; Conventional, EBV-negative and non-MSI-high

*P* values with statistically significant differences (< 0.05) are marked with an asterisk (*)

^a^*P* value between EBV-positive gastric carcinomas and conventional gastric carcinomas

^b^*P* value between MSI-high gastric carcinomas and conventional gastric carcinomas

EBV-positive gastric carcinomas had higher incidences of tumoral PD-L1(+) and immune cell PD-L1(+), and larger numbers of CD8^+^, FOXP3^+^, and PD-1^+^ TILs than conventional gastric carcinomas (*P* < 0.05 for all comparisons) (Table 3 and Supplemental Fig. 3). Specifically, tumoral PD-L1 and immune cell PD-L1 were expressed in 15 cases (47%) and 30 cases (94%), respectively, out of the 32 EBV-positive carcinomas. The CD8^+/high^, FOXP3^+/high^, and PD-1^+/high^ TILs were found in 30 (94% of EBV-positive subgroup), 21 (66%), and 23 (72%) cases, respectively. All 15 tumoral PD-L1(+)/EBV-positive carcinomas were enriched with CD8^+/high^ TILs.

**Table 3:** Comparison of PD-L1 Expression and Tumor-Infiltrating Lymphocytes in Each Molecular Group

	Conventional (*n* = 429)	EBV-positive (*n* = 32)	MSI-high (*n* = 53)
PD-L1 in tumor cells		*^a^*P* < 0.001	*^b^*P* < 0.001
Positive	59 (14%)	15 (47%)	27 (51%)
Negative	370 (86%)	17 (53%)	26 (49%)
PD-L1 in immune cells		*^a^*P* < 0.001	
Positive	186 (43%)	30 (94%)	28 (53%)
Absent	243 (57%)	2 (6%)	25 (47%)
CD8^+^		*^a^*P* < 0.001	*^b^*P* < 0.001
High	189 (44%)	30 (94%)	38 (72%)
Low	240 (56%)	2 (6%)	15 (28%)
FOXP3^+^		*^a^*P* = 0.036	
High	199 (46%)	21 (66%)	20 (38%)
Low	230 (54%)	11 (34%)	33 (62%)
PD-1^+^		*^a^*P* < 0.001	
High	142 (33%)	23 (72%)	16 (30%)
Low	287 (67%)	9 (28%)	37 (70%)

*EBV* Epstein-Barr virus, *MSI* Microsatellite instability; Conventional, EBV-negative and non-MSI-high

*P* values with statistically significant differences (< 0.05) are marked with an asterisk (*)

^a^*P* value between EBV-positive gastric carcinomas and conventional gastric carcinomas

^b^*P* value between MSI-high gastric carcinomas and conventional gastric carcinomas

MSI-high gastric carcinomas more commonly displayed tumoral PD-L1(+), immune cell PD-L1(+), or CD8^+/high^ TILs than conventional gastric carcinomas (*P* < 0.05, each) (Table 3 and Supplemental Fig. 3). Specifically, tumoral PD-L1(+), immune cell PD-L1(+), and CD8^+/high^ TILs were observed in 27 (51% of MSI-high carcinomas), 28 (53%), and 38 cases (72%), respectively.

### Prognostic value of PD-L1 and TILs in EBV-positive gastric carcinomas and MSI-high gastric carcinomas

**Fig. 4:**
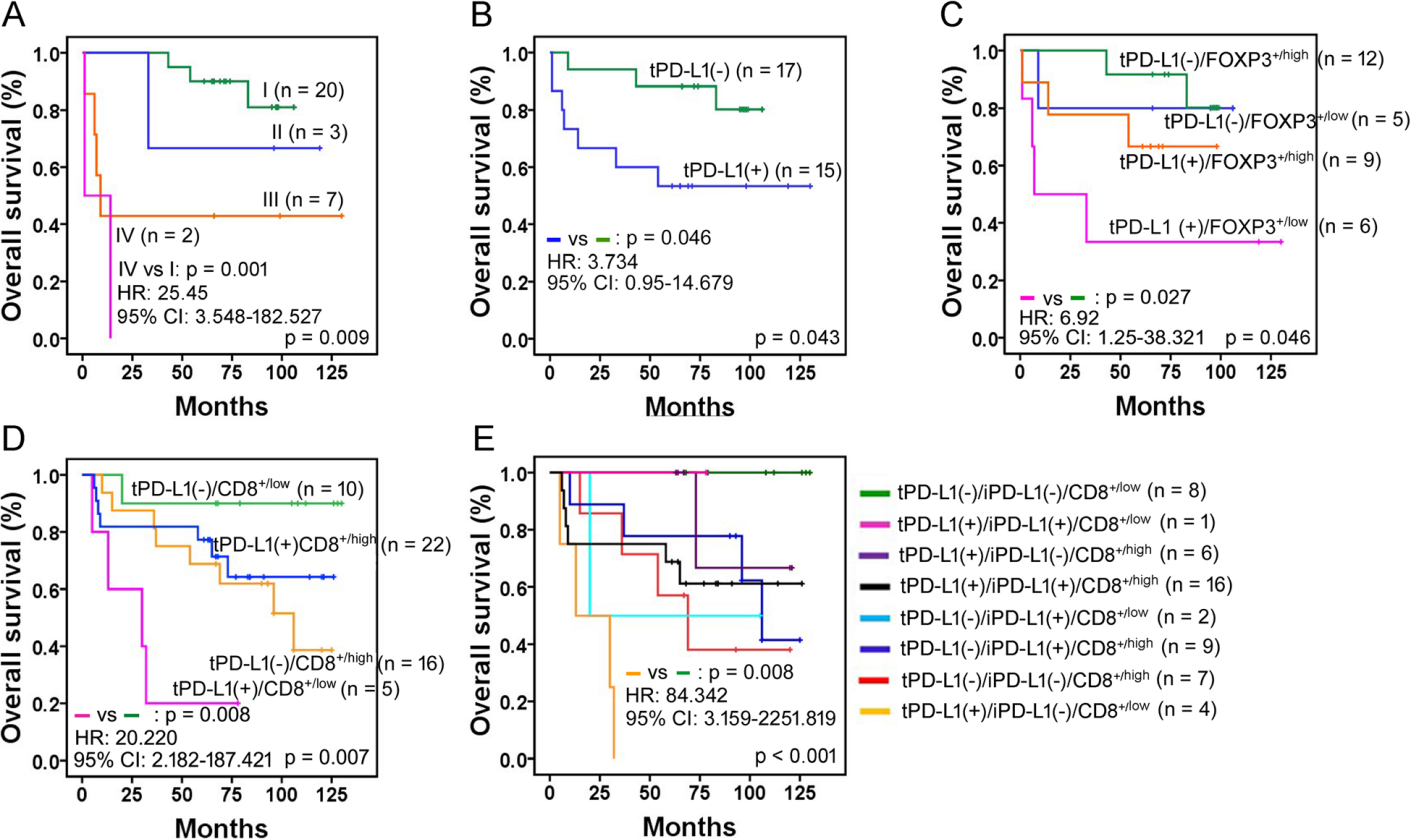
Kaplan-Meier plots of overall survival in EBV-positive or MSI-high gastric carcinomas. **a**-**c** In EBV-positive gastric carcinomas (*N* = 32), more advanced-stage tumors (**a**), tumoral PD-L1(+) (**b**), and the combined subset of tumoral PD-L1(+)/FOXP3^+/low^ TILs (**c**) are associated with lower rates of patient survival. I, II, III and IV in (**a**) indicate TNM tumor stage. **d**-**e** In MSI-high gastric carcinomas (*N* = 53), the combined subsets of tumoral PD-L1(+)/CD8^+/low^ TILs (**d**) and tumoral PD-L1(+)/immune cell PD-L1(−)/CD8^+/low^ TILs (**e**) show a worse overall survival rate. Corresponding *P* value, hazard ratio (HR) and 95% confidence interval (CI) in the worst prognostic subset are shown in the bottom left corner of each plot, and *P* values throughout all subsets, in the bottom right corner of each plot

In EBV-positive gastric carcinomas, the tumoral PD-L1(+) subgroup showed lower rates of overall survival (*P* < 0.05) (Fig. 4 and Supplemental Table 3), and recurrence-free survival (data not shown). Through further univariate analysis of tumoral PD-L1 status combined with each line of TILs, tumoral PD-L1(+)/FOXP3^+/low^ TILs revealed a worse clinical outcome (Fig. 4 and Supplemental Table 4); however, tumor stage (pTNM) was the only independent prognostic factor in EBV-positive gastric carcinomas. The prognostic significance of tumoral PD-L1(+)/CD8^+/low^ TILs could not be statistically evaluated in the EBV-positive group because all tumoral PD-L1(+)/EBV-positive cases contained CD8^+/high^ TILs (Supplemental Fig. 4).

In MSI-high gastric carcinomas, the combined subset of tumoral PD-L1(+)/CD8^+/low^ TILs or tumoral PD-L1(+)/immune cell PD-L1(−)/CD8^+/low^ TILs was associated with an adverse outcome for patients (*P* < 0.05, each) (Fig. 4). None of other parameters showed a prognostic impact on overall survival or recurrence-free survival through univariate analysis. Accordingly, multivariate analysis could not be performed reliably. The covariate parameters of multivariate analysis must include significant factors from univariate analysis, and factors containing the same parameter (tumoral PD-L1 in this situation) should not be considered together when doing multivariate analysis.

### Effects of PD-L1 knockdown in gastric carcinoma cells lines

**Fig. 5:**
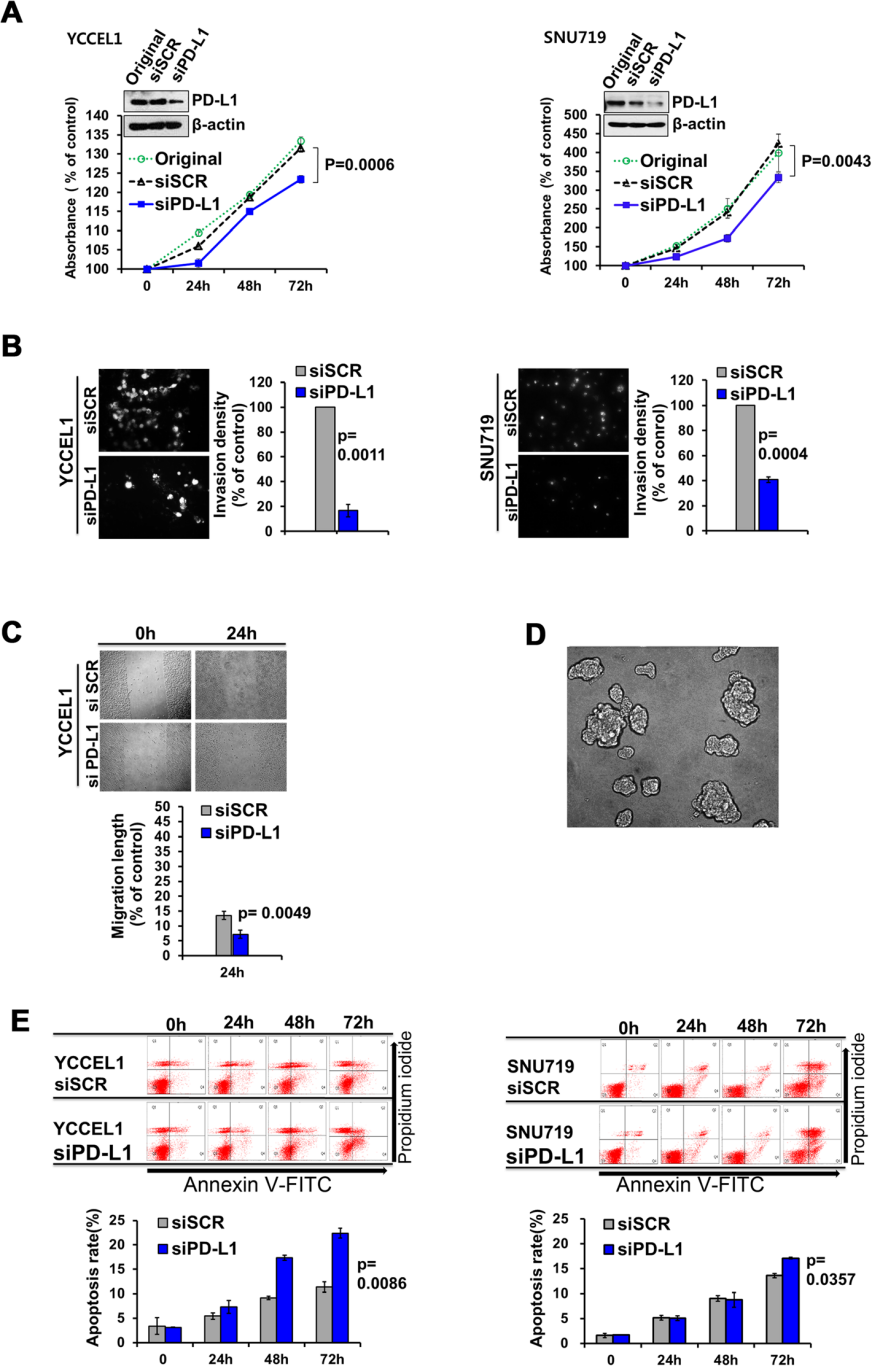
Effect of PD-L1 knockdown on cell biologic properties in EBV-positive gastric carcinoma cell lines. In YCCEL1 (left column) and SNU719 cells (right column), cell proliferation (**a**), invasion (**b**) and migration (**c**) are lower in PD-L1-specific siRNA transfected cells than scrambled siRNA-transfected cells, and apoptosis (**e**) is higher (*P* < 0.05). **d** There was a limitation on a wound-healing assay for migration in SNU719 cells; they grew in an aggregate pattern and thus, were not scratched with a pipette. X-axis denotes

**Fig. 6:**
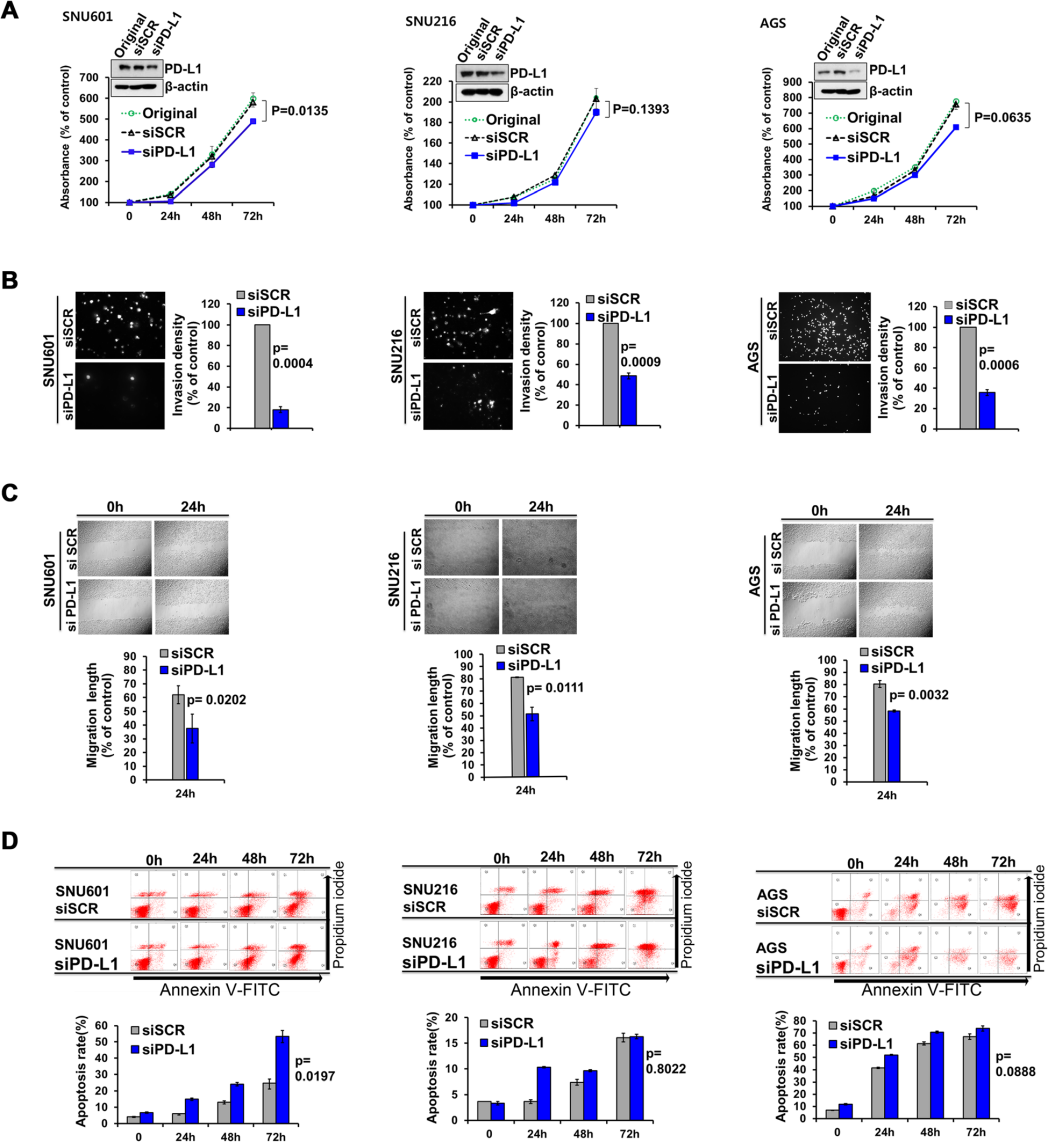
Effect of PD-L1 knockdown on cell biologic properties in EBV-negative gastric carcinoma cell lines. SNU 601 cells (in left column) show lower cell proliferation (**a**), invasion (**b**) and migration (**c**), and higher apoptosis (**d**) in PD-L1-specific siRNA cells than scrambled siRNA-transfected mock cells (*P* < 0.05). However, in SNU216 cells (in middle column) and AGS cells (in right column), PD-L1-specific siRNA cells reveal statistically significant decreases in invasion and migration compared to scrambled siRNA-transfected mock cells (*P* < 0.05), but not statistically significant changes in proliferation and apoptosis

Cell proliferation, invasion and migration were lower in PD-L1-specific siRNA transfected cells than in scrambled siRNA-transfected control cells, whereas apoptosis was higher. These findings were observed in all five gastric carcinoma cell lines; they were all statistically significant in two EBV-positive cell lines (Fig. 5), but not all in EBV-negative cell lines (Fig. 6). Specifically, SNU601 cells (EBV-negative) showed statistically significant differences with regard to proliferation, invasion, migration, and apoptosis, similar to the two EBV-positive cell lines. Our data imply that innate role of PD-L1 may be a facilitator of cancer cell survival.

## Discussion

The present study indicates that the prognostic value of PD-L1 is complex, and contingent on the tumor microenvironment, and EBV and MSI statuses. There has been few study analyzing combined three components of “tumoral PD-L1, immune cell PD-L1 and TILs” in a single study all together. In this study, the combined subset of “tumoral PD-L1(+)/immune cell PD-L1(-)/CD8^+/low^ TILs” predicted a worse clinical outcome and were an independent prognostic factor; however, individual PD-L1 or CD8^+^ TILs alone did not have an independent prognostic significance. In addition, CD8^+/low^ TILs was a worse prognostic factor in AGC group (Supplemental Fig. 2), specifically in tumor stage III (Supplemental Fig. 5) as assessed through univariate analysis. Unexpectedly, CD8^+/low^ TILs was correlated with well-known favorable prognostic factors such as Lauren intestinal type, less lymphatic invasion, and earlier tumor stage (Supplemental Table 5). In addition, tumoral PD-L1(+)/FOXP3^+/low^ TILs were associated with an unfavorable clinical outcome in the entire cohort, EBV-positive subgroup, and MSI-high subgroup through univariate analysis, while tumoral PD-L1(+)/immune cell PD-L1(−) status was associated with an unfavorable clinical outcome in the entire cohort only. Our combined analysis, which accounted for TILs in the tumor microenvironment, may provide reliable results regarding the prognostic value of PD-L1 expression. In this study, tumoral PD-L1 expression alone was an adverse prognostic factor, through univariate analysis, for all patients except those in the MSI-high gastric carcinoma subgroup. Our data on cell proliferation, invasion, migration and apoptosis using PD-L1 knockdown in gastric carcinoma cell lines demonstrated that the innate function of PD-L1 was to foster cancer cell survival, which is in line with prior studies [28, 29]. The exact mechanisms by which PD-L1 exerts innately oncogenic effects remain to be better defined. The underlying processes may be related to be that PD-L1 expression is intrinsically regulated via various oncogenic pathways as well as extrinsically mediated via cytokines in tumor microenvironment [30]. As a fundamental immunology concept, tumoral PD-L1 expression helps the tumor to evade host immune surveillance, possibly contributing to more severe outcomes in cancer patients. However, the prognostic impact of tumoral PD-L1 expression in gastric carcinomas has been contradictory; poor [12–15], good [16], and neutral [17] prognostic outcomes have all been reported. The reasons for these discordant results largely lie in the use of different antibodies and cut-off values in the evaluation of PD-L1 expression [31]. Further, we addressed additional possibilities for the reasons, namely the analysis of individual factors only, the analysis of a small series, or potential selection bias (non-consecutive cases). The present study is important because it included a combined analysis of PD-L1, multiple lines of TILs, and EBV-infection and MSI status together in the same cohort of a large series composed of consecutive surgically-resected gastric carcinomas.

The present report advocates that immune cell PD-L1 expression may predict a better clinical outcome. Overall, PD-L1(+) in immune cells were a favorable prognostic indicator. This may be because immune cell PD-L1 is regulated via an adaptive mechanism within the context of persistent tumor antigen-specific immune stimulation and reflects a pre-existing robust antitumor immunity, which may contribute to tumor surveillance and cytotoxic antitumor activity [32]. Similar to our results, previous studies have shown that PD-L1(+) in immune cells are associated with a favorable prognosis in gastric carcinomas [33–35]. In mouse models, PD-L1-expressing cytotoxic T cells seem to participate in antitumor immune responses via enhanced survival and potent expansion of cytotoxic T cells [36]. In contrast, PD-L1 in stromal immune cells has been shown to be involved in mediating the immune suppression of antitumor T cell responses [37]. Further studies are warranted to explain these contradictory data.

The present report suggests that FOXP3^+^ TILs may lean towards a good prognostic outcome. A possible explanation for our results is that FOXP3^+^ cells can suppress tumor-promoting proinflammatory cytokines that lead to malignancy [38]. This hypothesis is supported by our observation that a high population of FOXP3^+^ cells was prevalent in early-stage tumors, as reported in previous studies [39]. Currently, the prognostic role of FOXP3^+^ TILs in gastric carcinomas remains unanswered; a high population of FOXP3^+^ TILs has been associated with both a better [22, 40] and worse prognosis [41], or not a reliable marker [6]. There is an argument that FOXP3^+^ TILs may have heterogeneous properties that are affected by the tumor site, and possibly the molecular subtype, mirroring different contexts within different tumor microenvironments [42, 43].

In the present study, we propose that the extrinsic mechanism in tumoral PD-L1 expression may act more often in EBV-positive and MSI-high gastric carcinomas, indicating that PD-1/PD-L1 blockade immunotherapy may be advantageous in these patients. In our study, tumoral PD-L1(+)/ CD8^+/high^ TILs were more prevalent in the EBV-positive and MSI-high groups than in the conventional group. This suggests that PD-L1 expression in tumor cells develops via an extrinsic mechanism (i.e., adaptive immune resistance), in which tumoral PD-L1 expression is extrinsically derived in response to the expression of inflammatory cytokines (in particular, to interferon-γ that is released by CD8^+^ cytotoxic T cells) [44]. Furthermore, we found that diffuse expression of PD-L1 in a strong intensity was more frequently observed in the EBV-positive subgroup than in the conventional subgroup (13% versus 5% of tumoral PD-L1 expressing-cases, respectively). Thus, extrinsic and intrinsic mechanisms in tumoral PD-L1 expression may work together more commonly in EBV-positive gastric carcinomas. The amplification of chromosomal region 9p24.1, which includes the PD-L1 gene, to promote intrinsic expression of PD-L1 has been reported in 15% of EBV-positive gastric carcinomas [45]. Our results in the EBV-positive and MSI-high groups are consistent with those of previous reports: namely, the proportion of EBV-positive and MSI-high cases within the entire cohort, the more frequent tumoral PD-L1(+) and abundant CD8^+^ TILs in both subgroups, tumoral PD-L1(+) as an unfavorable prognostic indicator in the EBV-positive subgroup, and the mutually exclusive nature of EBV-positive and MSI-high status [12, 46, 47].

Our study has inherent limitations. First, our retrospective study merely represents immune context at the time of surgical resection, but not time-dependent dynamic immune heterogeneity. Second, we used only MLH1/MSH2 immunohistochemistry to screen cases for MSI multiplex PCR. We might miss out MSI cases with the isolated loss of PMS2 or MSH6, although those may be exceptional, at the least, in gastric carcinomas. Recently, Mathiak et al. reported that none of MSI-high gastric carcinomas has shown the isolated loss of MSH6 or PMS2 [48], which are more likely to be associated with Lynch syndrome due to a germline mutation in one of these genes [49]. Lastly, considering expression heterogeneity within the tumor, immunohistochemistry in as large a portion of tissue as possible, such as multiple full-sectioned tissues, would be better, but this is almost impractical. Instead, we utilized tissue microarray blocks composed of two tissue cores for each case in the deepest tumor invasion portion.

## Conclusions

The prognostic impact of PD-L1 is determined by the tumor microenvironment, and statuses of EBV and MSI. Tumoral or immune cell PD-L1 on their own are not independent prognostic factors, but they have a prognostic significance in certain molecular groups. Notably, the combination of PD-L1 expression and CD8^+^ TILs may serve as an independent prognostic factor. The combined subset of “tumoral PD-L1(+)/immune cell PD-L1(-)/CD8^+/low^ TILs” shows a worse prognosis. This may be advantageous for combinatorial therapies of PD-L1/PD-1 and cytotoxic T-lymphocyte associated antigen 4 blockades that would bring the activation and proliferation of effector T cells, thereby restoring the immune system toward attacking the tumor [50].

## Supplementary information

**Additional file 1: Supplemental Figure 1.** Correlation between PD-L1 expression and multiple lines of tumor-infiltrating lymphocytes (TILs). The tumoral PD-L1(+) subgroup (A-C) or immune cell PD-L1(+) subgroup (D-F) displays many more CD8^+^, FOXP3^+^, and PD-1^+^ TILs than each respective PD-L1(−) subgroup.

**Additional file 2: Supplemental Figure 2.** Kaplan-Meier plots of overall survival in advanced gastric carcinomas (AGC) (*n* = 253). Among advanced gastric carcinomas, subgroup containing tumors with CD8^+/high^ (A), and MSI-high subgroup (B) show more favorable outcomes than CD8^+/low^ or conventional subgroup, respectively. Corresponding *P* value, hazard ratio (HR) and 95% confidence interval (CI) in the worst prognostic subset are shown in the bottom left corner of each plot, and *P* values throughout all subsets, in the bottom right corner of each plot.

**Additional file 3: Supplemental Figure 3.** Comparison of the quantities of CD8^+^, FOXP3^+^, and PD-1^+^ TILs in the different molecular groups. CD8^+^ (A), FOXP3^+^ (B), and PD-1^+^ (C) TILs are more highly abundant in EBV-positive (EBV+) carcinomas than in conventional carcinomas. In MSI-high (MSI-H) carcinomas, only CD8^+^ TILs are significantly enriched compared to conventional carcinomas. * *P* < 0.05, ** *P* < 0.01, *** *P* < 0.001.

**Additional file 4: Supplemental Figure 4.** Distribution of tumoral PD-L1 combined with CD8^+^ population in each molecular subgroup. The EBV-positive and MSI-high (MSI-H) subgroups show a higher frequency of tumoral PD-L1(+)/CD8^+/high^ TILs than the conventional subgroup (*P* < 0.05, each).

**Additional file 5: Supplemental Figure 5.** Kaplan-Meir plots of overall survival in each tumor stage according to the density of CD8^+^ TILs. Corresponding *P* value, hazard ratio (HR) and 95% confidence interval (CI) in the worst prognostic subset are shown in the bottom left corner of each plot, and *P* values throughout all subsets, in the bottom right corner of each plot.

**Additional file 6: Supplemental Table 1.** Correlation Between PD-L1 Expression and Tumor-Infiltrating Lymphocytes (TILs) in the Entire Cohort (*N* = 514)

**Additional file 7: Supplemental Table 2.** Univariate and Multivariate Analyses in the Entire Cohort (*N* = 514) (corresponding to Fig. 2a, b & e)

**Additional file 8: Supplemental Table 3.** Univariate and Multivariate Analyses in EBV-positive Gastric Carcinomas (*n* = 32) (corresponding to Fig. 4a & c)

**Additional file 9: Supplemental Table 4.** Univariate Analysis in MSI-high Gastric Carcinomas (*n* = 53) (corresponding to Fig. 4d & e)

**Additional file 10: Supplemental Table 5.** Comparison of Clinicopathologic Characteristics, and Statuses of PD-L1, FOXP3^+^, and PD-1^+^ According to the Density of CD8^+^ TILs in the Entire Cohort (*N* = 514)

## Data Availability

Please contact the corresponding author for data requests.

## Abbreviations

AGC: Advanced gastric carcinoma
CTLA4: Cytotoxic T-lymphocyte associated antigen 4
EBV: Epstein-Barr virus
FOXP3: Forkhead box P3
MSI: Microsatellite instability
PD-1: Programmed death-1
PD-L1: Programmed death-ligand 1
pTNM: pathological tumor-node-metastasis
TIL: Tumor infiltrating lymphocyte
