# Supplementary figures and images for: Prognostic perspectives of PD-L1 combined with tumor-infiltrating lymphocytes, Epstein-Barr virus, and microsatellite instability in gastric carcinomas
Source: Diagn Pathol. 2020 Jun 4;15:69. doi: 10.1186/s13000-020-00979-z (PMC7271517; doi:10.1186/s13000-020-00979-z)

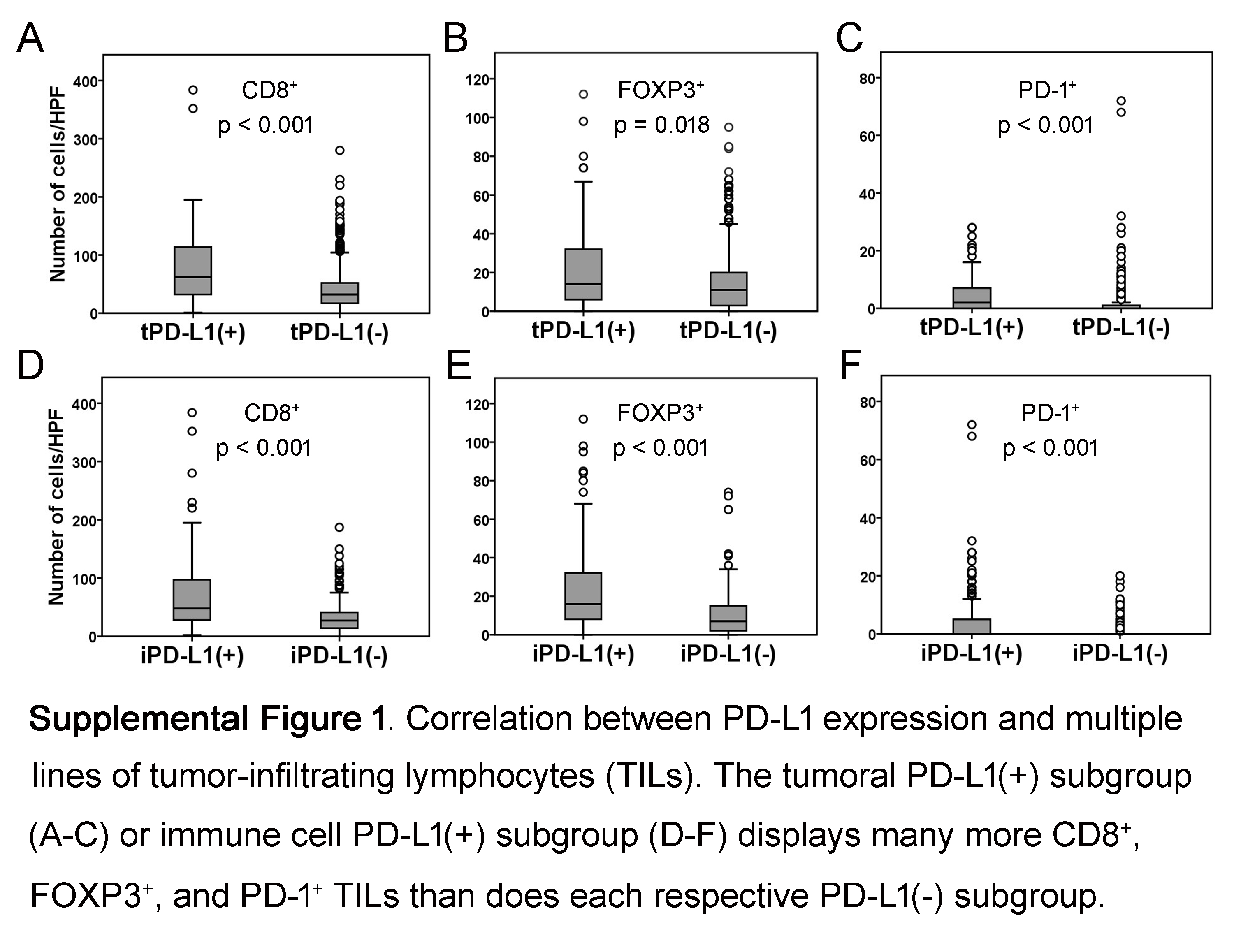

Supplement: Supplementary file 1 — Additional file 1: Supplemental Figure 1. Correlation between PD-L1 expression and multiple lines of tumor-infiltrating lymphocytes (TILs). The tumoral PD-L1(+) subgroup (A-C) or immune cell PD-L1(+) subgroup (D-F) displays many more CD8+, FOXP3+, and PD-1+ TILs than each respective PD-L1(−) subgroup. [file 13000_2020_979_MOESM1_ESM.tif]

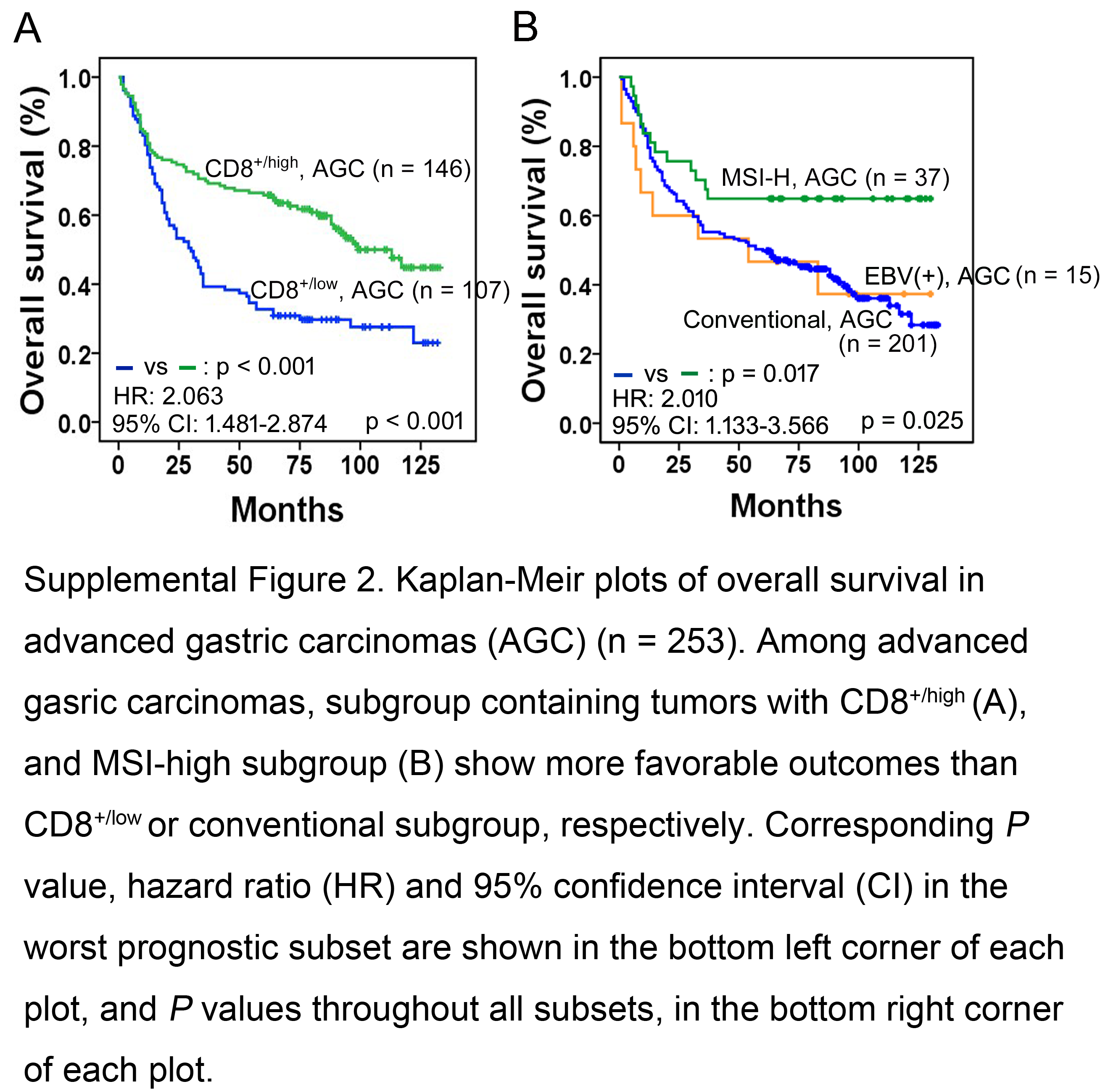

Supplement: Supplementary file 2 — Additional file 2: Supplemental Figure 2. Kaplan-Meier plots of overall survival in advanced gastric carcinomas (AGC) (n = 253). Among advanced gastric carcinomas, subgroup containing tumors with CD8+/high (A), and MSI-high subgroup (B) show more favorable outcomes than CD8+/low or conventional subgroup, respectively. Corresponding P value, hazard ratio (HR) and 95% confidence interval (CI) in the worst prognostic subset are shown in the bottom left corner of each plot, and P values throughout all subsets, in the bottom right corner of each plot. [file 13000_2020_979_MOESM2_ESM.tif]

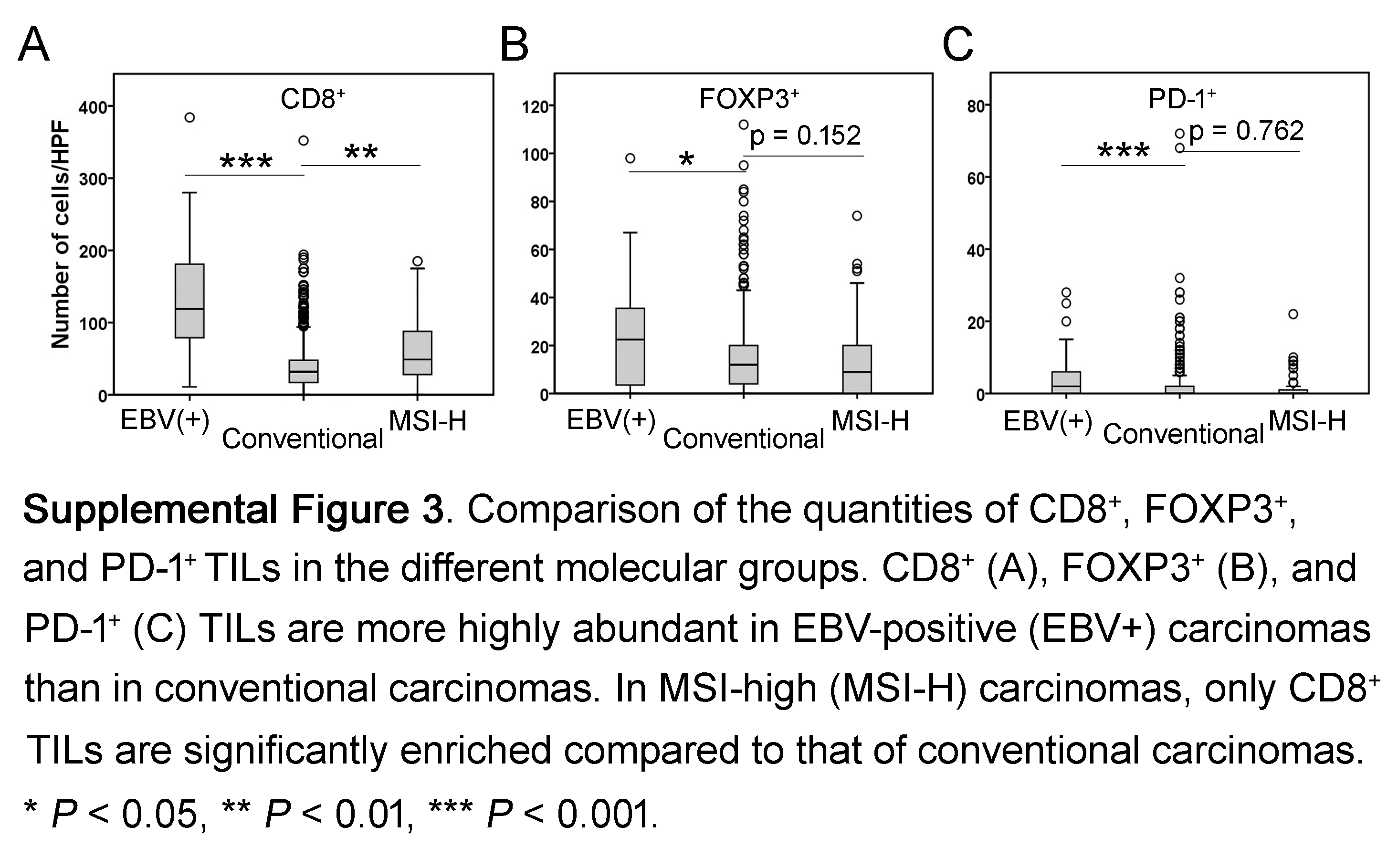

Supplement: Supplementary file 3 — Additional file 3: Supplemental Figure 3. Comparison of the quantities of CD8+, FOXP3+, and PD-1+ TILs in the different molecular groups. CD8+ (A), FOXP3+ (B), and PD-1+ (C) TILs are more highly abundant in EBV-positive (EBV+) carcinomas than in conventional carcinomas. In MSI-high (MSI-H) carcinomas, only CD8+ TILs are significantly enriched compared to conventional carcinomas. * P < 0.05, ** P < 0.01, *** P < 0.001. [file 13000_2020_979_MOESM3_ESM.tif]

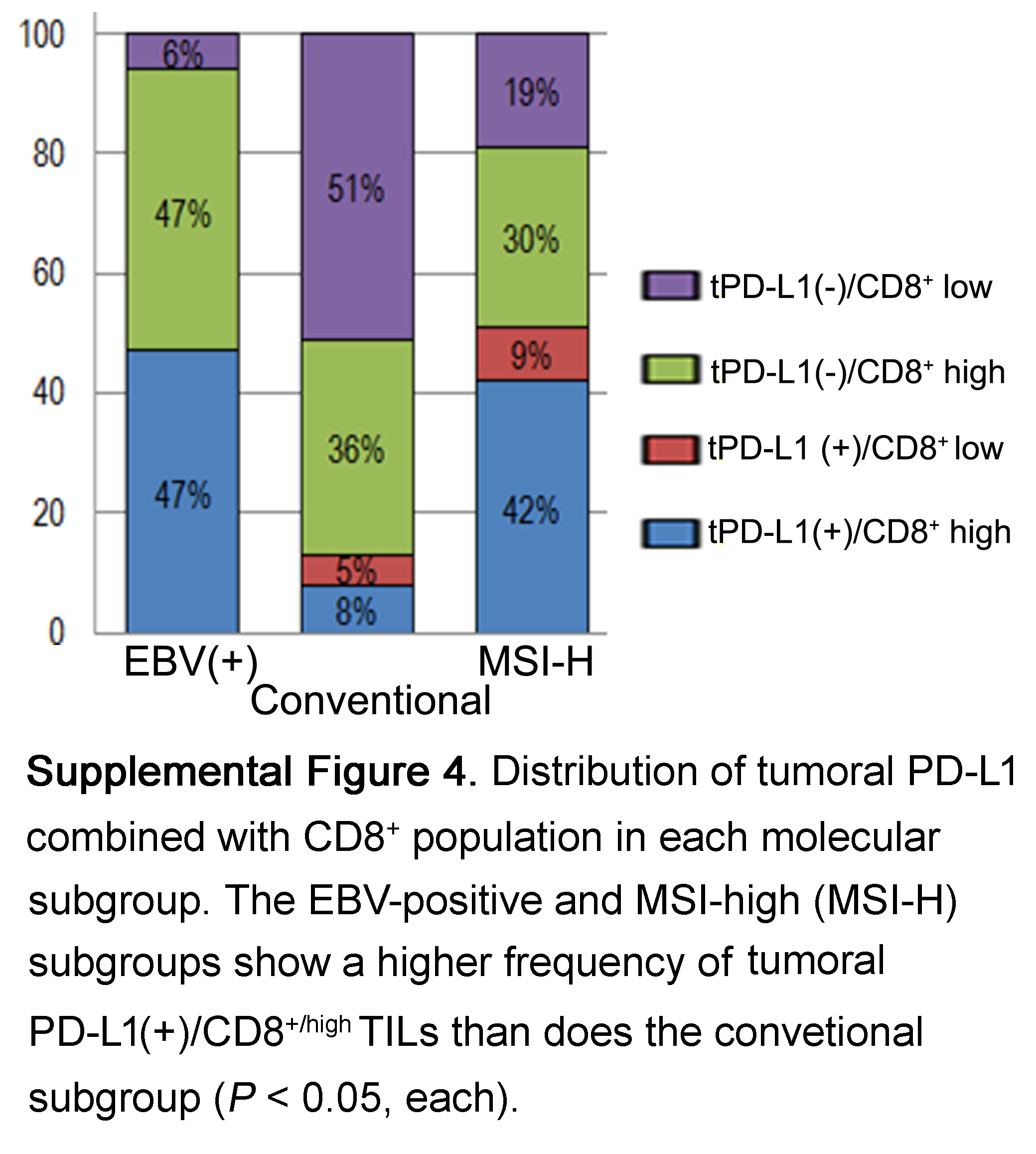

Supplement: Supplementary file 4 — Additional file 4: Supplemental Figure 4. Distribution of tumoral PD-L1 combined with CD8+ population in each molecular subgroup. The EBV-positive and MSI-high (MSI-H) subgroups show a higher frequency of tumoral PD-L1(+)/CD8+/high TILs than the conventional subgroup (P < 0.05, each). [file 13000_2020_979_MOESM4_ESM.tif]

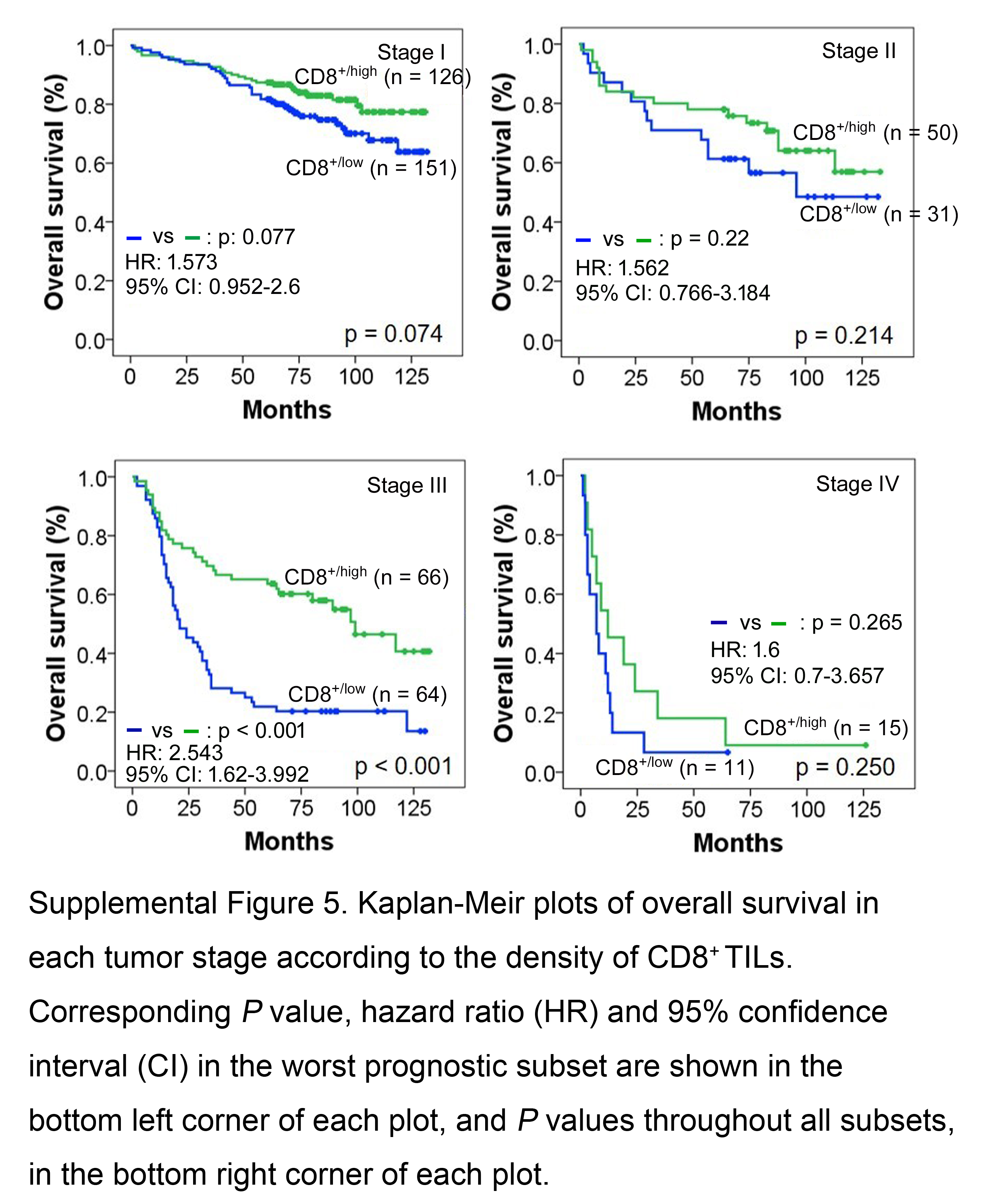

Supplement: Supplementary file 5 — Additional file 5: Supplemental Figure 5. Kaplan-Meir plots of overall survival in each tumor stage according to the density of CD8+ TILs. Corresponding P value, hazard ratio (HR) and 95% confidence interval (CI) in the worst prognostic subset are shown in the bottom left corner of each plot, and P values throughout all subsets, in the bottom right corner of each plot. [file 13000_2020_979_MOESM5_ESM.tif]
