# Supplementary material for: Prognostic perspectives of PD-L1 combined with tumor-infiltrating lymphocytes, Epstein-Barr virus, and microsatellite instability in gastric carcinomas
Source: Diagn Pathol. 2020 Jun 4;15:69. doi: 10.1186/s13000-020-00979-z (PMC7271517; doi:10.1186/s13000-020-00979-z)
Supplement: Supplementary file 6 — Additional file 6: Supplemental Table 1. Correlation Between PD-L1 Expression and Tumor-Infiltrating Lymphocytes (TILs) in the Entire Cohort (N = 514) [file 13000_2020_979_MOESM6_ESM.docx]

**Supplemental Table 1.** Correlation Between PD-L1 Expression and Tumor-Infiltrating Lymphocytes (TILs) in the Entire Cohort

|  | Total | PD-L1 in tumor cells | |
| --- | --- | --- | --- |
|  |  | positive | negative |
|  | (N = 514) | (N = 101) | (N = 413) |
| PD-L1 in immune cells |  | ** P* < 0.001 |  |
| positive | 244 (47%) | 65 (64%) | 179 (43%) |
| negative | 270 (53%) | 36 (36%) | 234 (57%) |
| CD8^+^ population |  | *** *P* < 0.001 |  |
| high | 257 (50%) | 73 (72%) | 184 (45%) |
| low | 257 (50%) | 28 (28%) | 229 (55%) |
| FOXP3^+^ population |  | *P* = 0.281 |  |
| high | 240 (47%) | 52 (51%) | 188 (46%) |
| low | 274 (53%) | 49 (49%) | 225 (54%) |
| PD-1^+^ population |  | *** *P* < 0.001 |  |
| high | 181 (35%) | 62 (61%) | 119 (29%) |
| low | 333 (65%) | 39 (39%) | 294 (71%) |

*P* values with statistically significant differences (< 0.05) are marked with an asterisk (*).
