# Supplementary material for: Prognostic perspectives of PD-L1 combined with tumor-infiltrating lymphocytes, Epstein-Barr virus, and microsatellite instability in gastric carcinomas
Source: Diagn Pathol. 2020 Jun 4;15:69. doi: 10.1186/s13000-020-00979-z (PMC7271517; doi:10.1186/s13000-020-00979-z)
Supplement: Supplementary file 7 — Additional file 7: Supplemental Table 2. Univariate and Multivariate Analyses in the Entire Cohort (N = 514) (corresponding to Fig. 2a, b & e) [file 13000_2020_979_MOESM7_ESM.docx]

**Supplemental Table 2.** Univariate and Multivariate Analyses in the Entire Cohort (N = 514) (corresponding to Fig. 2A, 2B & 2E)

| Variables | Categories | Univariate analysis |  | Multivariate analysis |  |
| --- | --- | --- | --- | --- | --- |
|  |  | Hazard ratio (95% CI) | *P* value | Hazard ratio (95% CI) | *P* value |
| pTNM tumor stage |  |  |  |  |  |
|  | II vs I | 1.947 (1.265-2.997) | 0.002* | 1.902 (1.176-3.076) | 0.009* |
|  | III vs I | 4.257 (3.057-5.927) | < 0.001* | 3.537 (2.265-5.522) | < 0.001* |
|  | IV vs I | 14.716 (9.078-23.856) | < 0.001* | 12.577 (6.911-22.889) | < 0.001* |
| tPD-L1/iPD-L1/CD8^+^ |  |  |  |  |  |
|  | tPD-L1(-)/iPD-L1(+)/CD8^+/low^  vs tPD-L1(-)/iPD-L1(+)/CD8^+/high^ | 1.284 (0.382-4.315) | 0.686 | 0.911 (0.265-3.125) | 0.882 |
|  | tPD-L1(+)/iPD-L1(+)/CD8^+/low^  vs tPD-L1(-)/iPD-L1(+)/CD8^+/high^  tPD-L1(-)/iPD-L1(+)/CD8^+/high^  vs tPD-L1(-)/iPD-L1(+)/CD8^+/high^ | 1.572 (0.850-2.907) | 0.149 | 0.780 (0.407-1.494) | 0.454 |
|  | tPD-L1(+)/iPD-L1(+)/CD8^+/high^  vs tPD-L1(-)/iPD-L1(+)/CD8^+/high^ | 1.036 (0.599-1.790) | 0.9 | 0.766 (0.439-1.339) | 0.35 |
|  | tPD-L1(-)/iPD-L1(-)/CD8^+/low^  vs tPD-L1(-)/iPD-L1(+)/CD8^+/high^ | 1.705 (1.042-2.790) | 0.034* | 1.128 (0.671-1.895) | 0.649 |
|  | tPD-L1(-)/iPD-L1(-)/CD8^+/high^  vs tPD-L1(-)/iPD-L1(+)/CD8^+/high^ | 1.829 (1.067-3.135) | 0.028* | 1.124 (0.635-1.990) | 0.688 |
|  | tPD-L1(+)/iPD-L1(-)/CD8^+/high^  vs tPD-L1(-)/iPD-L1(+)/CD8^+/high^ | 1.984 (0.877-4.488) | 0.1 | 0.675 (0.289-1.576) | 0.363 |
|  | tPD-L1(+)/iPD-L1(-)/CD8^+/low^  vs tPD-L1(-)/iPD-L1(+)/CD8^+/high^ | 4.716 (2.345-9.483) | < 0.001* | 2.548 (1.24-5.235) | 0.011* |
| tPD-L1/FOXP3+ |  |  |  |  |  |
|  | tPD-L1(+)/FOXP3^+/high^  vs tPD-L1(-)/FOXP3^+/high^ | 1.077 (0.598-1.939) | 0.806 | 0.739 (0.405-1.349) | 0.325 |
|  | tPD-L1(-)/FOXP3^+/low^  vs tPD-L1(-)/FOXP3^+/high^ | 1.629 (1.173-2.264) | 0.004* | 1.275 (0.907-1.791) | 0.162 |
|  | tPD-L1(+)/FOXP3^+/low^  vs tPD-L1(-)/FOXP3^+/high^ | 2.933 (1.877-4.582) | < 0.001* | 1.5 (0.938-2.400) | 0.09 |

CI, confidence interval

*P* values with statistically significant differences (< 0.05) are marked with an asterisk (*).
