# Supplementary material for: Prognostic perspectives of PD-L1 combined with tumor-infiltrating lymphocytes, Epstein-Barr virus, and microsatellite instability in gastric carcinomas
Source: Diagn Pathol. 2020 Jun 4;15:69. doi: 10.1186/s13000-020-00979-z (PMC7271517; doi:10.1186/s13000-020-00979-z)
Supplement: Supplementary file 8 — Additional file 8: Supplemental Table 3. Univariate and Multivariate Analyses in EBV-positive Gastric Carcinomas (n = 32) (corresponding to Fig. 4a & c) [file 13000_2020_979_MOESM8_ESM.docx]

**Supplemental Table 3.** Univariate and Multivariate Analyses in EBV-positive Gastric Carcinomas (n = 32) (corresponding to Fig. 4A & 4C)

| Variables | Categories | Univariate analysis |  | Multivariate analysis |  |
| --- | --- | --- | --- | --- | --- |
|  |  | Hazard ratio (95% CI) | *P* value | Hazard ratio (95% CI) | *P* value |
| pTNM tumor stage |  |  |  |  |  |
|  | II vs I | 2.348 (0.243-22.688) | 0.461 | 0.764 (0.032-18.283) | 0.868 |
|  | III vs I | 6.590 (1.455-29.846) | 0.014* | 2.784 (0.231-33.612) | 0.421 |
|  | IV vs I | 24.148 (3.392-171.907) | 0.001* | 25.45 (3.548-182.517) | 0.009* |
| tPD-L1/FOXP3^+^ |  |  |  |  |  |
|  | tPD-L1(-)/FOXP3^+/low^  vs tPD-L1(-)/FOXP3^+/high^ | 1.326 (0.12-14.629) | 0.818 | 1.519 (0.127-18.104) | 0.741 |
|  | tPD-L1(+)/FOXP3^+/high^  vs tPD-L1(-)/FOXP3^+/high^ | 2.587 (0.426-15.720) | 0.302 | 1.212 (0.088-16.658) | 0.886 |
|  | tPD-L1(+)/FOXP3^+/low^  vs tPD-L1(-)/FOXP3^+/high^ | 6.920 (1.250-38.321) | 0.027* | 5.417 (0.566-51.883) | 0.143 |

EBV, Epstein-Barr virus; CI, confidence interval

*P* values with statistically significant differences (< 0.05) are marked with an asterisk (*).
