# Supplementary material for: Prognostic perspectives of PD-L1 combined with tumor-infiltrating lymphocytes, Epstein-Barr virus, and microsatellite instability in gastric carcinomas
Source: Diagn Pathol. 2020 Jun 4;15:69. doi: 10.1186/s13000-020-00979-z (PMC7271517; doi:10.1186/s13000-020-00979-z)
Supplement: Supplementary file 9 — Additional file 9: Supplemental Table 4. Univariate Analysis in MSI-high Gastric Carcinomas (n = 53) (corresponding to Fig. 4d & e) [file 13000_2020_979_MOESM9_ESM.docx]

**Supplemental Table 4.** Univariate Analysis in MSI-high Gastric Carcinomas (n = 53) (corresponding to Fig. 4D & 4E)

| Variables | Categories | | Hazard ratio (95% CI) | *P* value |
| --- | --- | --- | --- | --- |
| tPD-L1/CD8^+^ | |  |  |  |
|  | | tPD-L1(+)/CD8^+/high^ vs tPD-L1(-)/CD8^+/low^ | 4.321 (0.528-35.392) | 0.173 |
|  | | tPD-L1(-)/CD8^+/high^ vs tPD-L1(-)/CD8^+/low^ | 5.971 (0.745-47.882) | 0.093 |
|  | | tPD-L1(+)/CD8^+/low^ vs tPD-L1(-)/CD8^+/low^ | 20.220 (2.182-187.421) | 0.008* |
| tPD-L1/iPD-L1/CD8^+^ | |  |  |  |
|  | | tPD-L1(+)/iPD-L1(-)/CD8^+/high^  vs tPD-L1(-)/iPD-L1(-)/CD8^+/low^ | 4.912 (0.146-165.269) | 0.375 |
|  | | tPD-L1(-)/iPD-L1(+)/CD8^+/high^  vs tPD-L1(-)/iPD-L1(-)/CD8^+/low^ | 9.419 (0.380-233.660) | 0.171 |
|  | | tPD-L1(+)/iPD-L1(+)/CD8^+/low^  vs tPD-L1(-)/iPD-L1(-)/CD8^+/low^ | 10.104 (0.134-761.711) | 0.294 |
|  | |  |  |  |
|  | | tPD-L1(+)/iPD-L1(+)/CD8^+/high^  vs tPD-L1(-)/iPD-L1(-)/CD8^+/low^ | 10.874 (0.457-258.771) | 0.140 |
|  | |  |  |  |
|  | | tPD-L1(-)/iPD-L1(-)/CD8^+/high^  vs tPD-L1(-)/iPD-L1(-)/CD8^+/low^ | 16.406 (0.656-410.263) | 0.088 |
|  | |  |  |  |
|  | | tPD-L1(-)/iPD-L1(+)/CD8^+/low^  vs tPD-L1(-)/iPD-L1(-)/CD8^+/low^ | 19.872 (0.583-677.560) | 0.097 |
|  | |  |  |  |
|  | | tPD-L1(+)/iPD-L1(-)/CD8^+/low^  vs tPD-L1(-)/iPD-L1(-)/CD8^+/low^ | 84.342 (3.159-2251.819) | 0.008* |

CI, confidence interval

*P* values with statistically significant differences (< 0.05) are marked with an asterisk (*).
