# Supplementary material for: Prognostic perspectives of PD-L1 combined with tumor-infiltrating lymphocytes, Epstein-Barr virus, and microsatellite instability in gastric carcinomas
Source: Diagn Pathol. 2020 Jun 4;15:69. doi: 10.1186/s13000-020-00979-z (PMC7271517; doi:10.1186/s13000-020-00979-z)
Supplement: Supplementary file 10 — Additional file 10: Supplemental Table 5. Comparison of Clinicopathologic Characteristics, and Statuses of PD-L1, FOXP3+, and PD-1+ According to the Density of CD8+ TILs in the Entire Cohort (N = 514) [file 13000_2020_979_MOESM10_ESM.docx]

**Supplemental Table 5.** Comparison of Clinicopathologic Characteristics, and Statuses of PD-L1, FOXP3^+^ and PD-1^+^ According to the Density of CD8^+^ TILs in the Entire Cohort (N = 514)

|  | CD8^+/high^ (n= 257) | CD8^+/low^ (n=257) | | *P* value |
| --- | --- | --- | --- | --- |
|  |  |  |  |  |
| Tumor site |  |  | | 0.224 |
| Lower 1/3 | 162 (63%) | 170 (66%) |  | |
| Middle 1/3 | 46 (18%) | 57 (22%) | |  |
| Upper 1/3 | 49 (19%) | 30 (12%) | |  |
| Histologic type |  |  | | 0.857 |
| Tubular adenocarcinoma | 209 (81%) | 206 (80%) | |  |
| Poorly cohesive carcinoma | 37 (14%) | 46 (18%) | |  |
| Mucinous carcinoma | 4 (2%) | 5 (2%) | |  |
| Undifferentiated carcinoma | 7 (3%) | 0 | |  |
| Lauren classification |  |  | | 0.004* |
| Intestinal | 127 (49%) | 159 (62%) | |  |
| Diffuse | 130 (51%) | 98 (38%) | |  |
| Lymphatic invasion |  |  | | 0.033* |
| Present | 127 (49%) | 103 (40%) | |  |
| Depth of invasion (pT) |  |  | | 0.006* |
| pT1 (mucosa, submucosa) | 112 (44%) | 150 (58%) | |  |
| pT2 (proper muscle) | 31 (12%) | 15 (6%) | |  |
| pT3 (subserosa) | 54 (21%) | 43 (17%) | |  |
| pT4 (serosa or beyond) | 60 (23%) | 49 (19%) | |  |
| Lymph node metastasis |  |  | | 0.421 |
| Present | 112 (44%) | 103(40%) | |  |
| Tumor stage (pTNM) |  |  | | 0.076 |
| I | 126 (49%) | 151 (59%) | |  |
| II | 50 (19%) | 31 (12%) | |  |
| III | 66 (26%) | 64 (25%) | |  |
| IV | 15 (6%) | 11 (4%) | |  |
| tPD-L1 |  |  | | < 0.001* |
| Positive | 73 (28%) | 28 (11%) | |  |
| iPD-L1 |  |  | | < 0.001* |
| Positive | 162 (63%) | 82 (32%) | |  |
| FOXP3^+^ |  |  | | 0.001* |
| High | 138 (54%) | 102 (40%) | |  |
| Low | 119 (46%) | 155 (60%) | |  |
| PD-1^+^ |  |  | | < 0.001* |
| High | 124 (48%) | 57 (22%) | |  |
| Low | 133 (52%) | 200 (78%) | |  |

*P* values with statistically significant differences (< 0.05) are marked with an asterisk (*).
